# *AANAT* transgenic sheep generated via OPS vitrified-microinjected pronuclear embryos and reproduction efficiency of the transgenic offspring

**DOI:** 10.7717/peerj.5420

**Published:** 2018-08-08

**Authors:** Xiuzhi Tian, Dongying Lv, Teng Ma, Shoulong Deng, Minghui Yang, Yukun Song, Xiaosheng Zhang, Jinglong Zhang, Juncai Fu, Zhengxing Lian, Shien Zhu, Yingjie Wu, Yiming Xing, Guoshi Liu

**Affiliations:** 1 National Engineering Laboratory for Animal Breeding, Key Laboratory of Animal Genetics and Breeding of the Ministry of Agriculture, Beijing Key Laboratory for Animal Genetic Improvement, College of Animal Science and Technology, China Agricultural University, Beijing, China; 2 Animal Genetic Resources Group, Institute of Animal Science, Chinese Academy of Agricultural Sciences, Beijing, China; 3 State Key Laboratory of Reproductive Biology, Institute of Zoology, Chinese Academy of Sciences, Beijing, China; 4 Tianjin Institute of Animal Sciences, Tianjin, China; 5 College of Biological Sciences, China Agricultural University, Beijing, China

**Keywords:** Sheep, Pronuclear microinjection, AANAT, OPS vitrification

## Abstract

**Background:**

The open pulled straw (OPS) vitrification method has been successfully applied in mouse, pig, and goat embryos as well as in buffalo oocytes, but it has not yet been applied to the microinjected embryos. This study examined the effects of OPS vitrification on embryo development and the reproductive capacity of the transgenic offspring in order to establish a method for preservation of microinjected embryos.

**Methods:**

Ovine pronuclear embryos were microinjected with the exogenous aralkylamine N-acetyltransferase gene (*AANAT*), frozen by the OPS method, and subsequently thawed for embryo transplantation. Pregnancy rate, lambing rate, survival rate, average birth weight and transgenic positive rate as well as reproduction efficiency and hormone level of the transgenic offspring were investigated to analyze the effect of OPS vitrification on microinjectd pronuclear embryos.

**Results:**

No significant differences were observed in the birth rate, lamb survival rate and transgenic positive rate between the frozen and non-frozen *AANAT*-microinjected pronuclear embryos. The average birth weight of the frozen embryos offspring was greater than that of the non-frozen embryos. Importantly, the transgenic offspring that overexpressed the *AANAT* gene showed improved ovulation efficiency and lambing rate by regulating their hormone levels.

**Conclusions:**

The OPS vitrification approach may be a valuable method in microinjected- embryo transfer technology, which could reserve embryos and result in fewer unnecessary animal sacrifices. In addition, the *AANAT*+ transgenic offspring exhibited improved reproductive capacity on account of regulation effect of melatonin on reproductive hormone. These data may provide available references for human-assisted reproduction.

## Introduction

Embryo freezing preserves embryos for transgenic use and allows embryo transfer (ET) to be unrestricted by time and distance. It simplifies the introduction process, prevents the spread of disease and provides technology to establish animal embryo libraries. Mouse embryos can be successfully frozen by different methods (Whittingham, Leibo & Mazur, 1972; Willadsen, 1977; Rall & Fahy, 1985). Vitrification provides high relatively freezing efficiency, and its application has successfully generated healthy offspring (Wilmut & Rowson, 1973; Willadsen et al., 1976; Hayashi et al., 1989; Kasiraj et al., 1993; Yokota et al., 2001; Wong & Wong, 2011). In 1997, the open pulled straw (OPS) method was reported. It achieved a 90% survival rate for bovine embryos produced in vitro as well as a 70% hatched blastocyst rate (Vajta et al., 1997a). The OPS method has been successfully applied in mouse, pig, and goat embryos (Cseh et al., 1997; Zhou et al., 2005; Vajta et al., 1997b; Holm et al., 1999; El-Gayar & Holtz, 2001), as well as in buffalo oocytes (Dhali et al., 2000).

Melatonin (N-acetyl-5-methoxytryptamine), a ubiquitously present and highly conserved molecule in many organisms, has multiple biological activities (Hardeland et al., 2012; Galano, Tan & Reiter, 2013). Serotonin N-acetyltransferase (aralkylamine N-acetyltransferase, *AANAT*) is the key rate-limiting enzyme in melatonin biosynthesis (Coon et al., 1995; Borjigin, Wang & Snyder, 1995; Klein et al., 1997). Previous studies have demonstrated that *AANAT* gene overexpression in algae, sheep, humans, and plants promoted melatonin biosynthesis. For example, *AANAT* transgenic tomatoes and rice exhibited significantly higher melatonin levels than did their wild-types (Okazaki et al., 2009; Park et al., 2013; Kang et al., 2010; Wang et al., 2014). In mammals, melatonin secreted by the pineal gland is important for regulating seasonal reproduction (Robinson et al., 1992a). Moreover, melatonin is also highly produced in normal human placenta and its synthetizing enzymes and receptors were confirmed expressing in human placenta throughout pregnancy, and reduced maternal plasma melatonin levels may be an early diagnostic tool to identify pregnancies complicated by preeclampsia, which suggested that melatonin played a beneficial role in placental function and pregnancy wellbeing (Lanoix, Guérin & Vaillancourt, 2012; Soliman et al., 2015). Melatonin affects sheep’s reproductive activity by regulating endocrine changes in the related hormones through the hypothalamus–pituitary–gonadal axis. This is mediated by melatonin receptors since this activity can be blocked by receptor antagonists. Many studies have confirmed the regulatory effects of exogenous melatonin on the estrous cycle and reproductive hormones of sheep (Robinson et al., 1992b; Mondain-Monval et al., 1988; Poulton et al., 1987). However, the effects of overexpressed *AANAT* on reproduction and reproductive hormones in sheep have not been reported.

Transgenic efficiency is an important factor in evaluating transgenic technology. Many investigators have successfully produced transgenic animals by pronuclear microinjection; however, the efficiency was considerably low. For example, the successful rates for pigs, miniature pigs, Merino sheep and goats were 1% (Hammer et al., 1985), 1.24% (Uchida et al., 2001), 0.47% (Murray et al., 1989), and 5% (Wang et al., 2002), relationship. The effects of a specific transgenic gene on vitrified-thawed microinjected pronuclear embryos have not been reported in any animals. In addition, whether freezing affects the transgenic efficiency or offspring development remains unclear. This study explored the survival rate of ovine pronuclear embryos microinjected with exogenous pBC1-*AANAT* after vitrification-thawing. Moreover, the pregnancy and lambing rates, as well as the positive rate of transgenic offspring, were monitored after embryo transplantation. Finally, the superovulation rate, pregnancy rate and average lamb survival numbers in the transgenic offspring were examined to explore the *AANAT* gene’s effect on reproduction efficiency.

## Materials and Methods

### Chemicals and reagents

Follicle-stimulating hormone (FSH), pregnant mare serum gonadotropin (PMSG), and human chorionic gonadotropin were purchased from Ningbo Hormone Products Co., Ltd (Ningbo, Zhengjiang, China). Melatonin and other reagents, unless specified, were purchased from Sigma Chemical Co. (Sigma Aldrich, St. Louis, MO, USA).

### Animals and ethics statement

All transgenic sheep production and sample collection procedures strictly followed the protocols approved by the Animal Welfare Committee of China Agricultural University (Permission Number: SYXK [Beijing] 2015002; the period of valid days: September 22, 2015–September 22, 2020), and the study was conducted in strict accordance with the guidelines and regulations established by this committee. The experimental ewes were White Suffolk, aged 1–4 years, without reproductive diseases and fed on a selected experimental farm for at least 1 month to achieve a healthy condition (55–90 kg). The rams weighed 75–85 kg and had a good reproductive drive and high-quality semen.

### AANAT gene expression vector construction

Total RNA was extracted from the pineal gland of the White Suffolk using Trizol Reagent (Invitrogen Inc., Carlsbad, CA, USA) per the manufacturer’s instructions. The extracted RNA was immediately used for cDNA synthesis with a first-strand cDNA synthesis kit (TaKaRa Bio Inc., Tokyo, Japan) per the manufacturer’s instructions. The *AANAT* gene coding sequence (CDS) was amplified using TransStart FastPfu DNA Polymerase (TransGen Biotech, Beijing, China). The specific primers (forward: 5′-CCGCTCGAGCCACCATGTCCACGCCAAGC-3′, backward: 5′-CCGCTCG AGCCACCTCAGCGGTCACTGTT-3′) were designed by Primer Premier 5.0 and added to the Xho I restriction sites per the *AANAT* sequence (mRNA KC290949.1, protein AGG68821.1). The mammalian expression vector, pBC1 (Invitrogen Inc., Carlsbad, CA, USA), was used as a backbone to prepare the gene constructs. *AANAT* was excised from the T-vector by Xho I and subsequently cloned into pBC1. The recombinant vector was referred to as pBC1-*AANAT*. The linearized DNA was digested by Sal I and Not I, then extracted from the gel and purified by a DNA Purification Kit (Tiangen, Beijing, China).

### Cas9 mRNA and sgRNA preparation

Cas9 and sgRNA plasmids were constructed per the previously published method (Han et al., 2014). Cas9 and sgRNA coding regions containing a T7 promoter were amplified by PCR using TransStart FastPfu DNA Polymerase (TransGen Biotech, Beijing, China) from each plasmid. The T7-Cas9 and T7-sgRNA polymerase chain reaction (PCR) products were purified and used as the template for in vitro transcription (IVT) using the mMESSAGE mMACHINE T7 ULTRA Transcription Kit (Life Technologies, Inc., Grand Island, NY, USA). The poly (A) tailing reaction was performed after the capping was complete using a Poly (A) Tailing Kit (Life Technologies, Inc., Grand Island, NY, USA) per the manufacturer’s instructions. After the IVT, the sgRNA and Cas9-encoding mRNA were purified by ethanol and lithium chloride separately, then precipitated and dissolved in RNase-free water, and finally stored at −80 °C until use.

### Superovulation and artificial insemination of donor sheep

Ewe estrous cycles were synchronized by a 14-day treatment of progesterone with a controlled internal drug release (CIDR) device containing 300 mg progesterone (EAZI-BREED^®^ CIDR^®^ Sheep and Goats Device Pfizer Animal Health, New Zealand). The donor ewes were given consecutive injections of 45, 45, 40, 35, 35, 30, and 30 IU of FSH (Ningbo Hormone Products CO., Ltd, Ningbo, Zhengjiang, China) every 12 h, and the CIDR was removed with the last injection. Superovulation in the donors was induced by injecting 320 IU of PMSG (Ningbo Hormone Products CO., Ltd, Ningbo, Zhengjiang, China) 12 h after sponge removal. For the recipients, their CIDRs were removed 10 h in advance of the donor’s CIDR withdrawal, and the recipients were injected with a single dose of 280–320 IU PMSG. A total of 24 h after CIDR withdrawal, all ewes were exposed at 6-h intervals to a ram wearing a cloth to detect estrous onset. Artificial insemination was performed using semen freshly collected by an artificial vagina from five rams, and diluted 1:1–4 with sheep semen diluent (glucose 31 g/L, sodium citrate 14 g/L, and neomycin sulfate 1 g/L). The endoscope (Karl Storz Endoskope GmbH, Tuttlingen, Germany) intrauterine horn method was used to inseminate donor ewes with 0.2–0.5 mL of fresh semen from five different rams (motility > 0.6) at the 54th h after CIDR removal.

### Embryo collection

All sheep were fasted for 12 h before embryo recovery to facilitate surgery and reduce post-operative intestinal adhesions. Pronuclear embryos were laparoscopically removed from the fallopian tubes 12 h after insemination. Embryos were collected by oviduct flushing with a cannula attached to a syringe and inserted into the lumen near the uterotubal junction. The fallopian tube was flushed with 20 mL of non-frozen solution (PBS-0.3% BSA) (Fraktion, 735078; Roche Diagnostics GmbH, Mannheim, Germany). The recovered flush solution was observed by stereomicroscope (SZ61; Olympus, Kawasaki, Japan) to search for embryos. The embryos were transferred into holding medium (Immuno-Chemical Products Ltd, Auckland, New Zealand) and qualitatively evaluated with an inverted microscope (IX71; Olympus, Kawasaki, Japan). The number of corpora lutea (CL) and the total number of recovered embryos were recorded for each ewe.

### Embryo microinjection

The acquired pronuclear embryos were immediately microinjected and cultured in vitro at 38.5 °C in a chamber with 5% CO_2_ and a humidified atmosphere within 30 min. Microinjection was performed with an inverted microscope equipped with a pair of micro manipulators (Narishige, Tokyo, Japan). Fertilized eggs were microinjected with two to five pL of Tris-EDTA (TE) solution containing 50 ng/μL of sgRNA and 100 ng/μL of Cas9 mRNA, then linearized DNA solution was injected with five pL of pBC1-*AANAT* at a concentration of 10 ng/μL. After microinjection, four to six embryos were transplanted into the recipient oviducts within 2 h, and the remaining embryos were vitrified for further study.

### Embryo vitrification and thawing

Embryos were frozen by the OPS method. First, approximately five embryos were pretreated in equilibration solution (ES) solution (10% ethylene glycol (EG) and 10% dimethyl sulfoxide (DMSO) in PBS) for 30 s, then transferred into the EDFS30 solution (15% EG, 15% DMSO, 18% (w/v) Ficoll (MW, 70,000) and 0.3 M sucrose in PBS). Finally, they were picked up by the narrow end of the OPS within 25 s and immediately plunged into liquid nitrogen. Embryos were removed from the liquid nitrogen to thaw, and the narrow tip was immersed in one mL of 0.5 M sucrose. Embryos were expelled from the OPS with a mouth pipette and immediately transferred into another drop of one mL of 0.5 M sucrose for 5 min to dilute the cryoprotectants at 37 °C. Recovered embryos were washed three times and evaluated under a stereomicroscope, and the surviving embryos (6–10 per recipient) were transplanted into the recipient oviducts within 1 h.

### Embryo transfer

Microinjected embryos were transplanted into recipients whose CIDRs had been removed 76 h prior. The recipient’s reproductive tract was exteriorized with minimal manipulation to assess the CL number and quality. An ET tube (Agtech, Inc., Manhattan, KS, USA) was attached to a one mL syringe to suck out the microinjected pronuclear embryos, then the ET tube was penetrated through the oviduct umbrella approximately three to four cm into the oviduct ampulla. Embryos in the holding medium at the front of the transfer catheter were deposited into the oviduct ipsilaterally through the fimbria.

### Pregnancy diagnosis and lambing

After ET, recipient pregnancies were detected by ultrasonography on the 60th day. Ultrasonic equipment (SonoVet 600 scanner; Universal Ultrasound, New York, NY, USA) with a five MHz linear array probe was used to conduct the diagnosis. Pregnancy was diagnosed by identifying at least one fetal image.

From the 146th day of pregnancy, ewes were kept indoors (two ewes/pen) to monitor the lambing initiation. Newborn lambs were weighed within 6 h after birth.

### Transgenic positive lamb screening

Genomic DNA was extracted from the ears of the offspring to determine their genotype (Dneasy Tissue Kit; Qiagen, Inc., Mississauga, ON, Canada). The transgenic gene was analyzed by Genome PCR. The forward primer AA8-F product for *AANAT* was located in the insertion sequence (forward: 5′-GTCCAGCACTTCCTGACCCT-3′, backward: 5′-CATCAGAAGTTAAA CAGCACAGTTAG-3′). PCR was performed as described by Ma et al. (2017). Products were sequenced by Sangon Biotech, Beijing, and blasted in the GenBank database.

### Hormone analysis

Animals were treated with a vaginal CIDR for 10 days, and blood was collected from the jugular vein at 0, 12, 24, 36, 48, and 60 h after vaginal CIDR removal. The blood was clotted for 30 min, then centrifuged at 850×g for 10 min to obtain serum. The serum was separated from the blood and stored at −80 °C prior to hormone analysis. Serum FSH, GnRH, E_2_, luteinizing hormone (LH), and melatonin (MT) levels were measured by radioimmunoassay (RIA) per the instructions for the FSH, GnRH, E_2_, LH, and MT Direct RIA Kits (ICN Biomedicals, Irvine, CA, USA). The RIAs chromatographically separated the steroids to enhance specificity and used a tracer with the charcoal-dextran method to separate the bound and free steroids. The kits included a specific antibody that had no significant cross-reactivity with other steroids. The melatonin assay method was performed as described by Zhao et al. (2013). All procedures were performed in an ice bath and kept in the dark. Samples were collected at night.

### Data analysis

Data are expressed as means ± SEM and were analyzed by AVOVA followed by Student’s *t*-test using GraphPad Prism (San Diego, CA, USA). *P*-values < 0.05 were considered statistically significant.

## Results

**Figure 1:**
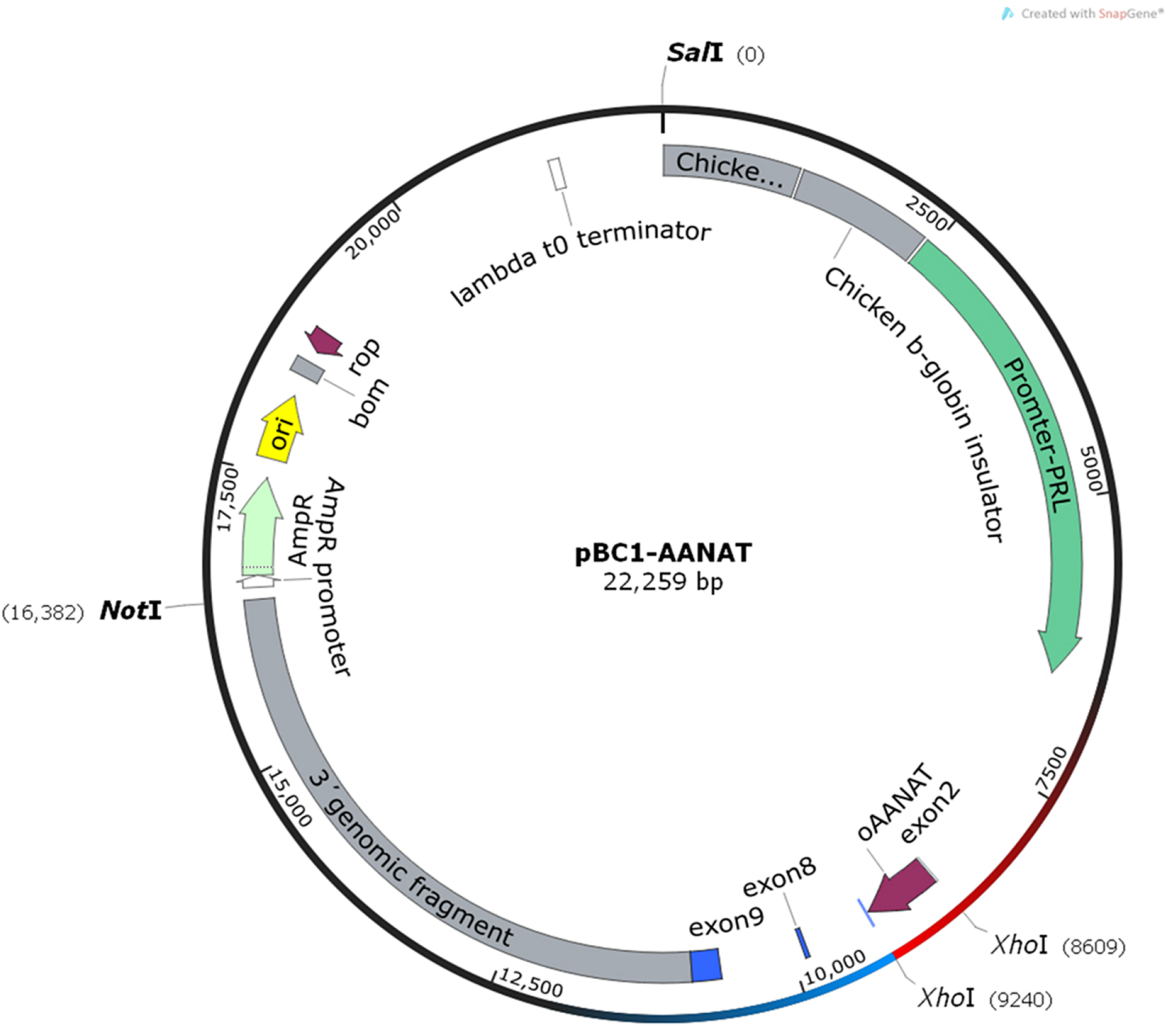
The structure of pBC1-AANAT plasmid.

Total RNA was extracted from the White Suffolk ewes’ pineal glands, and the first-strand cDNA was synthesized. The intact *AANAT* CDS was amplified, and the Xho I restriction site was added (634 bp full-length). The corrected cDNA was verified by Sanger sequencing, excised from the T-vector by Xho I, and subsequently cloned into the mammary gland-specific expression vector, pBC1 (Invitrogen, Inc., Carlsbad, CA, USA). The recombinant vectors were referred to as pBC1-*AANAT*. The gene was driven by a β-casein promoter to produce the recombinant protein (Fig. 1).

**Figure 2:**
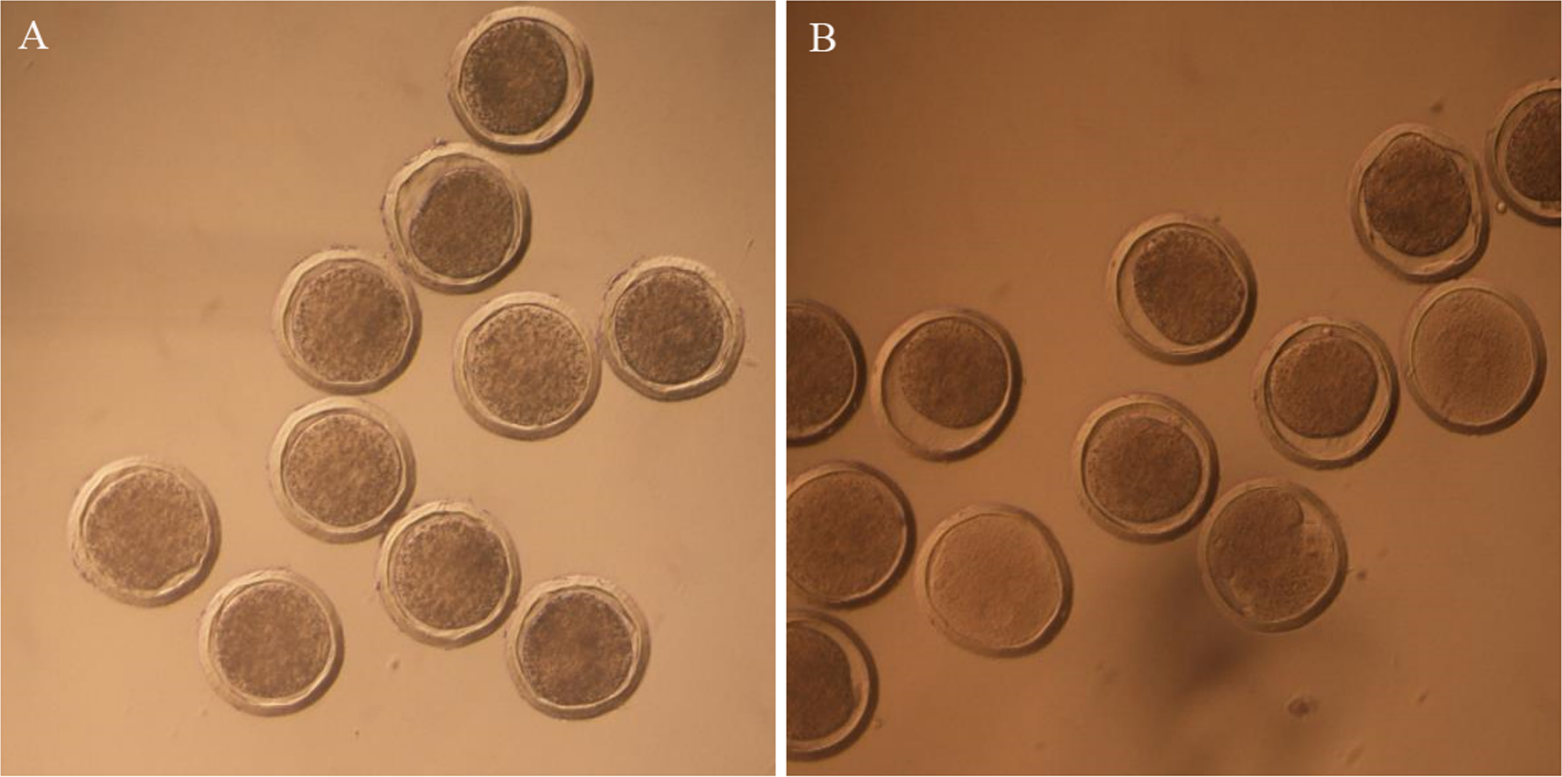
Pictures of embryos before and after vitrification. (A) Microinjected embryos before vitrification; (B) microinjected embryos after thawing.

Sixty-three donors were used to produce non-frozen microinjected pronuclear embryos. A total of 1,007 embryos were collected, and 855 were microinjected and transplanted into 167 recipients after 30–60 min of the microinjection, yielding a utilization rate of 98.5% (855/868). The pregnancy rate was 33.5% (56/167), and 74 offspring were born, yielding a lambing rate of 44.3% (74/167). After birth, 67 of the 74 lambs survived and the survival rate was 90.5% (67/74). On the other hand, nine donors were used to produce frozen microinjected pronuclear embryos. The 139 embryos were collected and 137 embryos were microinjected and experienced for OPS vitrification. After thawing, 122 embryos were viable and the survival rate was 89.1% (122/137). The viable embryos presented uniform cytoplasmic and smooth zona pellucida (Fig. 2). Finally, the vitrified-microinjected pronuclear embryos were transplanted into 14 recipients. The pregnancy rate was 21.4% (3/14), and five offspring were born; thus, the lambing rate was 35.7% (5/14), and the lambing survival rate was 80.0% (4/5). The average birth weight of the non-frozen and frozen groups were 3.6 and 4.4 kg, respectively. The pregnancy and lamb survival rates of the frozen embryos were lower than those of the non-frozen embryos, while the average birth weight was reversed between the two groups (Table 1).

**Table 1:** Embryo transfer efficiencies of frozen/non-frozen microinjected pronuclear embryos and the positive rate of the offspring.

Group	No. of recipients	No. of embryos transferred	Pregnancy rate (%)	Lambing rate (%)	No. of survival offspring	Survival rate (%)	No. of average lamb survival	The average birth weight (kg)	PCR positive rate(%)	Squencing positive rate (%)
Non-frozen	167	5.1^a^	33.5^a^ (56/167)	44.3^a^ (74/167)	67	90.5^a^ (67/74)	1.2 (67/56)	3.6^b^	63.7 (47/74)	63.7 (47/74)
Frozen	14	8.7^b^	21.4^b^ (3/14)	35.7^b^ (5/14)	4	80.0^b^ (4/5)	1.3 (4/3)	4.4^a^	60.0 (3/5)	60.0 (3/5)

**Notes:**

Each value represents the mean. Different superscripts in the same column differ significantly (*P* < 0.05). Pregnancy rate: no. of pregnancy recipients/No. of recipients transferred; Lambing rate: no. of offspring/No. of recipients transferred; survival rate: no. of survival offspring/No. of offspring; No. of average lamb survival: no. of survival offspring/no. of pregnancy recipients.

**Figure 3:**
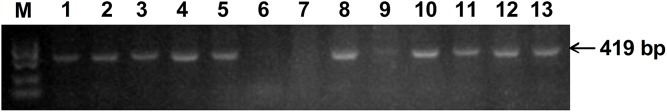
The PCR detection of transgenic offspring. M, marker, 1–13 represents transgenic offspring.

**Figure 4:**
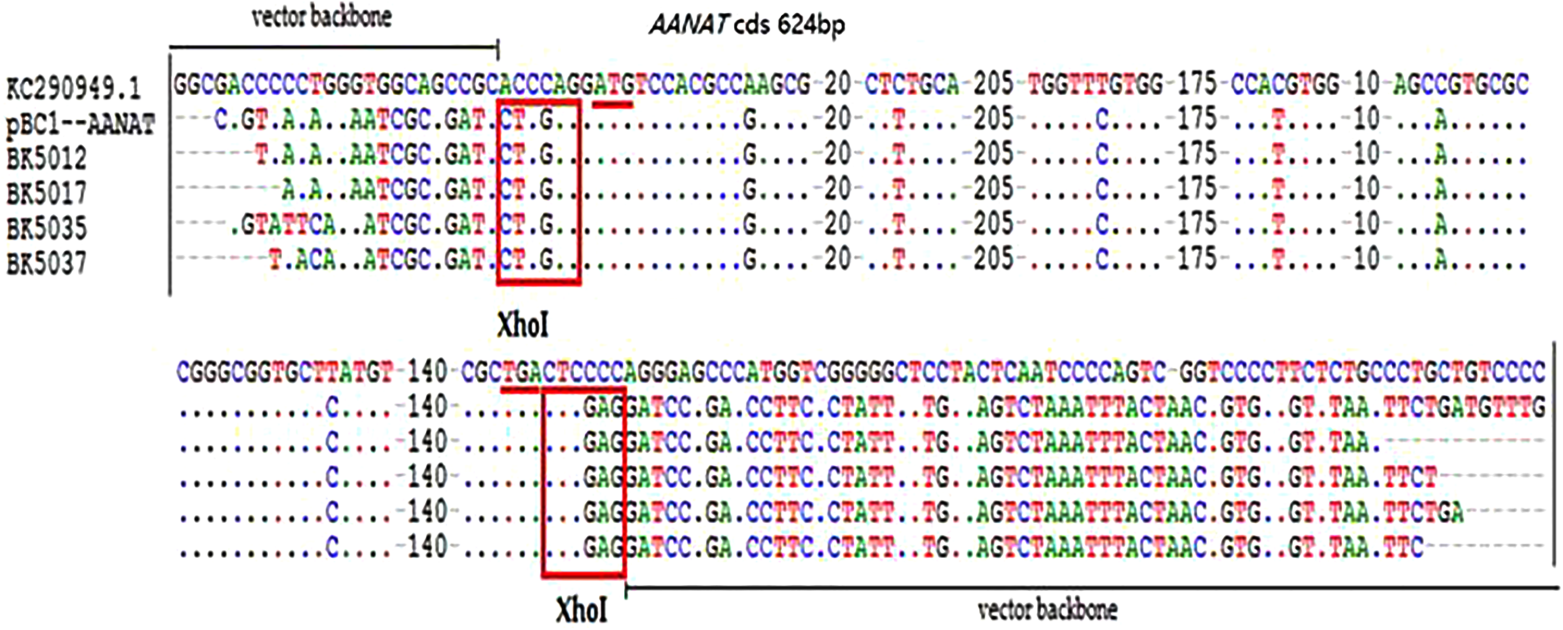
The sequencing detection of transgenic offspring.

To improve the CRISPR/Cas9 system integration efficiency, the sgRNA-targeted myostatin (*MSTN*) gene was designed (Ma et al., 2017). The mixture of mRNA from Cas9/sgRNA and DNA from *AANAT* was injected into the pronuclear embryo cytoplasm. In the non-frozen group, 47 of the 74 lambs were transgenic-positive, and in the frozen group, three of five lambs were transgenic-positive. All transgenic-positive lambs were identified by sequencing. The transgenic-positive rates were 63.5% and 60% in the non-frozen and frozen groups, respectively. The transgenic-positive rate did not significantly differ between the two groups (Table 1; Figs. 3 and 4).

The superovulation efficiency of the transgenic vs. non-transgenic offspring was investigated. Those offspring come from non-frozen microinjected pronuclear embryos. The offspring overexpressed *AANAT* ovulated significantly more oocytes than the *AANAT-*negative offspring (15.0 ± 2.6 vs. 8.4 ± 1.5) (Table 2).

**Table 2:** Superovulation efficiency of the transgenic offspring.

Group	No. of donors	No. of average acquired oocytes
*AANAT*−	7	8.4 ± 1.5^b^
*AANAT*+	3	15.0 ± 2.6^a^

**Notes:**

Each value represents the mean. Different superscripts in the same column differ significantly (*P* < 0.01).

The reproductive efficiency of the transgenic offspring showed that the pregnancy rate and the average number of surviving lambs in the *AANAT-*overexpressed offspring were higher than those of the *AANAT-*negative offspring (57.9% vs. 42.1%; 1.8 vs. 1.2, respectively), while the average birth weight of the *AANAT-*overexpressed offspring did not significantly differ compared to the non-transgenic offspring (3.7 ± 0.3 vs. 3.5 ± 0.7 kg) (Table 3).

**Table 3:** Reproductive efficiency of the transgenic and non-transgenic offspring.

Group	Pregnancy rate (%)	No. of average lamb survival	The average birth weight (kg)
*AANAT*−	42.1^b^ (8/19)	1.2^b^	3.5 ± 0.7
*AANAT*+	57.9^a^ (11/19)	1.8^a^	3.7 ± 0.3

**Notes:**

Each value represents the mean.

Different superscripts in the same column differ significantly (*P* < 0.01).

**Figure 5:**
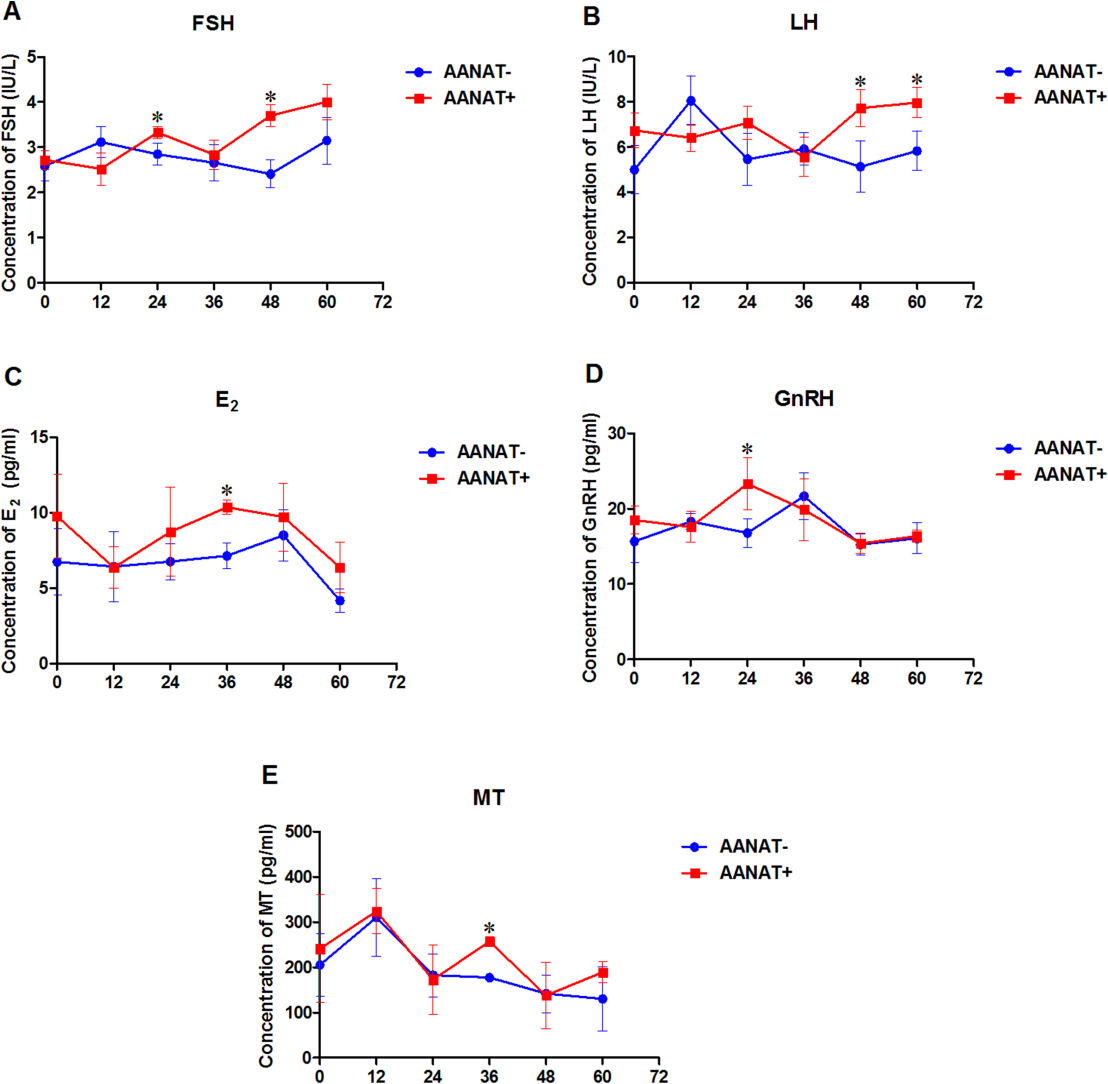
Serum FSH, LH, E_2_, GnRH, and MT were measured in *AANAT*+ and *AANAT−* females. *AANAT*+: *AANAT* transgenic-positive females; *AANAT−*: AANAT-negative females. (A–E) Serum production of FSH, LH, E_2_, GnRH, and MT collected at 0, 12, 24, 36, 48, and 60 h after vaginal CIDR removal from the control and transgenic-positive females. The times of 12, 36, and 60 h were at night. *Represents significant differences, *P* < 0.05.

Serum FSH production from the *AANAT+* females was higher than that in the *AANAT-* females at 24, 48, and 60 h following the vaginal CIDR; however, the FSH level was significantly higher at 24 and 48 h after the vaginal CIDR was removed in the *AANAT+* than in the *AANAT*− sheep. LH production was similar to FSH production in both groups. Serum E_2_ in the *AANAT+* females was higher than that in the *AANAT*− females at 24, 48, and 60 h following vaginal CIDR removal and was significantly higher at 36 h. GnRH levels in the *AANAT+* females were significantly higher than in *AANAT*− females 24 h after vaginal CIDR removal, while they were lower than in *AANAT*− females for the remaining time. Most importantly, MT levels at night (collected 36 and 60 h after vaginal CIDR removal at 3:00 am) were higher in the *AANAT+* females than in the *AANAT*− females, the *AANAT+* females exhibited stronger melatonin circadian rhythmicity than that of *AANAT*− counterparts. During the day, melatonin levels in both groups were comparable (Fig. 5).

## Discussion

Few reports were on the influence of OPS vitrification and thawing on microinjected pronuclear embryo quality in mammals, particularly in ovine. In the current study, the *AANAT* transgenic-positive rate of the offspring from vitrified-thawed embryos did not significantly differ from the non-frozen microinjected pronuclear embryos. This indicates that OPS vitrification had minimal adverse effects on embryo development and could serve as a valuable method in microinjected ovine pronuclear embryo transplantation technology. The use of OPS method would reserve embryos and result in fewer unnecessary animal sacrifices. Survival rates in mouse pronuclear embryos subjected to OPS freezing were shown to be significantly lower than those of the morulae (Zhou et al., 2005). The reduced survival rate may have resulted from free radical formation and ice crystals in frozen embryos (Bank & Brockbank, 1987). In our study, the survival rate (89.1%) was significantly higher than that previously reported (Zhou et al., 2005). This difference might be species-specific, but it also strongly suggests that overexpressed the *AANAT* gene in the embryos may exert some protective effects. Elevated melatonin levels are known to effectively protect vitrified-thawed embryo development (Reiter et al., 2016). By reducing apoptosis and reactive oxygen species (ROS) in bovine somatic cell nuclear transfer embryos, melatonin enhanced blastocyst quality and ultimately improved bovine cloning efficiency (Su et al., 2015). In mice, the pregnancy rates for non-frozen eight cells and blastocysts were found to be 40% and 100%, while the pregnancy rates for frozen eight cells and blastocysts were 36.4% and 90%, respectively. In humans, pregnancy rates for frozen pronuclear embryos were 45.5%, and the offspring survival rate was 20.9% (Wong & Wong, 2011). In the current study, the pregnancy rate was 21.4% and the offspring survival rate was 80%. These differences were species-specific as the litter size order is mice > sheep > humans. Short-term storage of microinjected embryos at 4 °C was reported to induce developmental arrest in rabbit embryos; however, no differences were reported in pregnancy rate, litter size and transgenic integration rate between non-frozen and frozen embryos (Nishijima et al., 2013). The pregnancy rate in this study was 33.5% in the non-frozen and 21.3% in the frozen groups, which is lower than that reported in domestic pigs (38–62%) (Pursel et al., 1990), miniature pigs (70%) (Uchida et al., 2001) and goats (45%) (Wang et al., 2002), but similar to that of Merino sheep (21–36%) (Murray et al., 1989). These results suggest that transgenic efficiencies are both species- and method-dependent. It appears that the microinjected pronuclear embryos can be successfully transferred after vitrification and can lead to successful pregnancies and relatively high rates of transgenic-positive offspring. Hence, the OPS vitrification approach on microinjected embryos may be a valuable method of ET to avoid wasting embryos and to synchronize recipients, thus reducing unnecessary animal sacrifices (Zhao et al., 2013). In addition, the mean birth weight of the offspring was significantly higher in the frozen group than that in the non-frozen group (4.4 kg vs. 3.6 kg, *P* < 0.05), this was consistent with an observation of human neonates (Vergouw et al., 2012). These results indicated that storing embryos at low temperatures may unknowingly impact offspring growth and development.

The published literature revealed that efficiency rates for generating transgenic animals by microinjection were approximately 1.0, 1.24, 0.47 and 5% in domestic pigs, miniature pigs, Merino sheep and goats, respectively (Hammer et al., 1985; Uchida et al., 2001; Murray et al., 1989; Wang et al., 2002). In our study, the transgenic efficiency in ovines was 2.19% (3/137) for the frozen embryos and 5.50% (47/855) for the non-frozen embryos, which was higher than those of other reports. Another important observation in the current study was that the reproductive functions, including superovulation, pregnancy rate and average number of surviving lambs in the transgenic offspring with overexpression of *AANAT* were improved compared to their non-transgenic counterparts. This has not been reported previously. These results were similar to those of previous reports on melatonin-treated embryos (Tian et al., 2017; Wang et al., 2013). The average numbers of oocytes between *AANAT*− and *AANAT+* differed significantly, with 15.0 oocytes from the *AANAT+* offspring, which is similar to the young sheep’s 15.1 (Zhang et al., 2013). Moreover, the pregnancy rate and average number of surviving lambs between *AANAT*− and *AANAT*+ offspring also differed significantly. We realized that the serum melatonin levels of both groups in most of the evaluated time points had no significant difference; however, it was reported that the serum melatonin only reflected the pineal gland originated melatonin but not the specific tissue melatonin levels (Hardeland, 2017). Thus, it cannot rule out the high level of melatonin in the reproductive system of *AANAT*+ offspring. We can assume that the *AANAT* gene was expressed and melatonin biosynthesis was elevated in the embryos, consequently reducing embryo damage induced by microinjection and freezing (Byeon & Back, 2016). Melatonin has potent protective effects on embryo quality, especially under cold stress (Jang et al., 2010). Furthermore, melatonin also significantly elevates serum estradiol-17β (E_2_) content, which may be mediated by MT2 activation (Jia et al., 2016). This study demonstrated that overexpressed *AANAT* promoted reproductive ability by advancing FSH and E_2_ levels.

## Conclusions

In conclusion, these results confirmed that OPS vitrification and thawing can be used to preserve microinjected pronuclear embryos, particularly in ovines, since this procedure had few adverse effects on the pronuclear embryos and resulted in a relatively high rate of offspring survival. Potential overexpression of the *AANAT* gene in embryos appeared to protect them from OPS vitrification and thawing by elevating melatonin biosynthesis and reducing ROS. The *AANAT* transgenic offspring exhibited improved reproductive capacity compared to their non-transgenic counterparts. This may relate to regulative effects of melatonin on reproductive hormone production. These data may provide references for human-assisted reproduction.

## Supplemental Information

10.7717/peerj.5420/supp-1 Supplemental Information 1 Raw data.

## Additional Information and Declarations

### Data Availability

The following information was supplied regarding data availability: The raw data are provided in a Supplemental File.
