# Supplementary material for: AANAT transgenic sheep generated via OPS vitrified-microinjected pronuclear embryos and reproduction efficiency of the transgenic offspring
Source: PeerJ. 2018 Aug 8;6:e5420. doi: 10.7717/peerj.5420 (PMC6087419; doi:10.7717/peerj.5420)
Supplement: Supplemental Information 1 [file peerj-06-5420-s001.zip › Raw data/Detection of MT and hormone/Detection of FSH.docx]

|  | **research data report of Beijing north institute of biotechnology co. LTD.** | | | | | | |
| --- | --- | --- | --- | --- | --- | --- | --- |
| data | 20171028 | | | | | | |
| Applicant Name | China Agricultural University | | | | | | |
| consignor | Xiuzhi Tian | | | contact information |  | | |
|  |  |  |  | E-MAIL |  | | |
| test item | FSH | | | | | | |
| Reagent batches | 20171020 | | | | | | |
| The reagent manufacturer | Beijing north institute of biotechnology co. LTD. | | | | | | |
| detection method | The standard products and samples were labeled with the antibody and incubated at 4℃ for 20hr, and then added separation agent and centrifuged for 15min at 3800rpm | | | | | | |
| instrument | Xi 'an nuclear instrument factory xh6080 radio-immunity analyzer | | | | | | |
| results |  |  |  |  |  |  |  |
| T | 32135 | 32135 |  | 0 |  |  |  |
| N | 1256 | 1256 | 1256 | 3.9 |  | r= | -0.99212 |
| 0 | 16489 | 16489 | 15233 | 47.4 |  | a= | 2.9 |
| 2.5 | 14904 | 14904 |  | 89.6 |  | b= | -2.42 |
| 5 | 12978 | 12978 |  | 77 |  |  |  |
| 10 | 9840 | 9840 |  | 56.4 |  | ed75= | 5.56 |
| 25 | 6371 | 6371 |  | 33.6 |  | ed50= | 15.78 |
| 50 | 4754 | 4754 |  | 23 |  | ed25= | 44.79 |
| 100 | 3532 | 3532 |  | 14.9 |  |  |  |
| ＱＣL： | 12287 |  |  | 72.4 |  |  | 6.31 |
| ＱＣH： | 6426 |  |  | 33.9 |  |  | 29.75 |
| numerical order |  |  |  |  |  |  | measurement value (mIU/ml) |
| 1 | 13301 |  |  | 79.1 |  |  | 4.46 |
| 2 | 13806 |  |  | 82.4 |  |  | 3.64 |
| 3 | 13456 |  |  | 80.1 |  |  | 4.21 |
| 4 | 15088 |  |  | 90.8 |  | L | 1.79 |
| 5 | 14521 |  |  | 87.1 |  | L | 2.57 |
| 6 | 14343 |  |  | 85.9 |  | L | 2.84 |
| 7 | 13770 |  |  | 82.2 |  |  | 3.69 |
| 8 | 13718 |  |  | 81.8 |  |  | 3.79 |
| 9 | 15127 |  |  | 91.1 |  | L | 1.73 |
| 10 | 14897 |  |  | 89.5 |  | L | 2.06 |
| 11 | 14139 |  |  | 84.6 |  |  | 3.13 |
| 12 | 13981 |  |  | 83.5 |  |  | 3.38 |
| 13 | 14093 |  |  | 84.3 |  |  | 3.2 |
| 14 | 15108 |  |  | 90.9 |  | L | 1.77 |
| 15 | 14612 |  |  | 87.7 |  | L | 2.44 |
| 16 | 14881 |  |  | 89.4 |  | L | 2.08 |
| 17 | 14392 |  |  | 86.2 |  | L | 2.77 |
| 18 | 13752 |  |  | 82 |  |  | 3.74 |
| 19 | 14152 |  |  | 84.7 |  |  | 3.11 |
| 20 | 14579 |  |  | 87.5 |  | L | 2.49 |
| 21 | 14058 |  |  | 84 |  |  | 3.27 |
| 22 | 14954 |  |  | 89.9 |  | L | 1.98 |
| 23 | 13766 |  |  | 82.1 |  |  | 3.71 |
| 24 | 14436 |  |  | 86.5 |  | L | 2.7 |
| 25 | 13897 |  |  | 83 |  |  | 3.5 |
| 26 | 14338 |  |  | 85.9 |  | L | 2.84 |
| 27 | 14325 |  |  | 85.8 |  | L | 2.86 |
| 28 | 13766 |  |  | 82.1 |  |  | 3.71 |
| 29 | 14835 |  |  | 89.1 |  | L | 2.15 |
| 30 | 14588 |  |  | 87.5 |  | L | 2.49 |
| 31 | 15055 |  |  | 90.6 |  | L | 1.84 |
| 32 | 14494 |  |  | 86.9 |  | L | 2.62 |
| 33 | 13767 |  |  | 82.1 |  |  | 3.71 |
| 34 | 14988 |  |  | 90.1 |  | L | 1.94 |
| 35 | 14256 |  |  | 85.3 |  | L | 2.97 |
| 36 | 15617 |  |  | 94.3 |  | L | 1.1 |
| 37 | 14506 |  |  | 87 |  | L | 2.59 |
| 38 | 15170 |  |  | 91.3 |  | L | 1.69 |
| 39 | 14511 |  |  | 87 |  | L | 2.59 |
| 40 | 14233 |  |  | 85.2 |  | L | 2.99 |
| 41 | 13867 |  |  | 82.8 |  |  | 3.55 |
| 42 | 14230 |  |  | 85.2 |  | L | 2.99 |
| 43 | 14877 |  |  | 89.4 |  | L | 2.08 |
| 44 | 14542 |  |  | 87.2 |  | L | 2.55 |
| 45 | 13670 |  |  | 81.5 |  |  | 3.86 |
| 46 | 14483 |  |  | 86.8 |  | L | 2.64 |
| 47 | 14034 |  |  | 83.9 |  |  | 3.29 |
| 48 | 13390 |  |  | 79.7 |  |  | 4.31 |
| 49 | 14312 |  |  | 85.7 |  | L | 2.88 |
| 50 | 14833 |  |  | 89.1 |  | L | 2.15 |
| 51 | 14031 |  |  | 83.9 |  |  | 3.29 |
| 52 | 13871 |  |  | 82.8 |  |  | 3.55 |
| 53 | 13804 |  |  | 82.4 |  |  | 3.64 |
| 54 | 14565 |  |  | 87.4 |  | L | 2.51 |
| 55 | 14652 |  |  | 87.9 |  | L | 2.4 |
| 56 | 14761 |  |  | 88.7 |  | L | 2.23 |
| 57 | 13986 |  |  | 83.6 |  |  | 3.36 |
| 58 | 14149 |  |  | 84.6 |  |  | 3.13 |
| 59 | 14242 |  |  | 85.2 |  | L | 2.99 |
| 60 | 15437 |  |  | 93.1 |  | L | 1.33 |
| 61 | 13830 |  |  | 82.5 |  |  | 3.62 |
| 62 | 14019 |  |  | 83.8 |  |  | 3.31 |
| 63 | 14047 |  |  | 84 |  |  | 3.27 |
| 64 | 14774 |  |  | 88.7 |  | L | 2.23 |
| 65 | 14886 |  |  | 89.5 |  | L | 2.06 |
| 66 | 13819 |  |  | 82.5 |  |  | 3.62 |
| 67 | 14536 |  |  | 87.2 |  | L | 2.55 |
| 68 | 13583 |  |  | 80.9 |  |  | 4.01 |
| 69 | 14482 |  |  | 86.8 |  | L | 2.64 |
| 70 | 14285 |  |  | 85.5 |  | L | 2.93 |
| 71 | 13584 |  |  | 80.9 |  |  | 4.01 |
| 72 | 14033 |  |  | 83.9 |  |  | 3.29 |
| 73 | 14278 |  |  | 85.5 |  | L | 2.93 |
| 74 | 14605 |  |  | 87.6 |  | L | 2.46 |
| 75 | 15865 |  |  | 95.9 |  | L | 0.79 |
| 76 | 14584 |  |  | 87.5 |  | L | 2.49 |
| 77 | 14103 |  |  | 84.3 |  |  | 3.2 |
| 78 | 14224 |  |  | 85.1 |  |  | 3.02 |
| 79 | 13762 |  |  | 82.1 |  |  | 3.71 |
| 80 | 14666 |  |  | 88 |  | L | 2.38 |
| 81 | 15157 |  |  | 91.3 |  | L | 1.69 |
| 82 | 14410 |  |  | 86.4 |  | L | 2.73 |
| 83 | 14474 |  |  | 86.8 |  | L | 2.64 |
| 84 | 14648 |  |  | 87.9 |  | L | 2.4 |
| 85 | 14832 |  |  | 89.1 |  | L | 2.15 |
| 86 | 15408 |  |  | 92.9 |  | L | 1.37 |
| 87 | 13974 |  |  | 83.5 |  |  | 3.38 |
| 88 | 13649 |  |  | 81.4 |  |  | 3.88 |
| 89 | 14108 |  |  | 84.4 |  |  | 3.18 |
| 90 | 14533 |  |  | 87.2 |  | L | 2.55 |
| 91 | 12479 |  |  | 73.7 |  |  | 5.93 |
| 92 | 14214 |  |  | 85.1 |  |  | 3.02 |
| 93 | 13891 |  |  | 82.9 |  |  | 3.52 |
| 94 | 14756 |  |  | 88.6 |  | L | 2.25 |
| 95 | 15048 |  |  | 90.5 |  | L | 1.86 |
| 96 | 14773 |  |  | 88.7 |  | L | 2.23 |
| 97 | 13820 |  |  | 82.5 |  |  | 3.62 |
| 98 | 13644 |  |  | 81.3 |  |  | 3.91 |
| 99 | 15335 |  |  | 92.4 |  | L | 1.47 |
| 100 | 14877 |  |  | 89.4 |  | L | 2.08 |
| 101 | 14245 |  |  | 85.3 |  | L | 2.97 |
| 102 | 13605 |  |  | 81.1 |  |  | 3.96 |
| 103 | 13638 |  |  | 81.3 |  |  | 3.91 |
| 104 | 14452 |  |  | 86.6 |  | L | 2.68 |
| 105 | 13619 |  |  | 81.2 |  |  | 3.93 |
| 106 | 13978 |  |  | 83.5 |  |  | 3.38 |
| 107 | 13387 |  |  | 79.6 |  |  | 4.33 |
| 108 | 13634 |  |  | 81.3 |  |  | 3.91 |
| 109 | 13012 |  |  | 77.2 |  |  | 4.96 |
| 110 | 14680 |  |  | 88.1 |  | L | 2.36 |
| 111 | 10965 |  |  | 63.7 |  |  | 9.25 |
| 112 | 14082 |  |  | 84.2 |  |  | 3.22 |
| 113 | 13315 |  |  | 79.2 |  |  | 4.43 |
| 114 | 13380 |  |  | 79.6 |  |  | 4.33 |
| 115 | 13370 |  |  | 79.5 |  |  | 4.36 |
| 116 | 13887 |  |  | 82.9 |  |  | 3.52 |
| 117 | 14188 |  |  | 84.9 |  |  | 3.06 |
| 118 | 13747 |  |  | 82 |  |  | 3.74 |
| 119 | 13718 |  |  | 81.8 |  |  | 3.79 |
| 120 | 13540 |  |  | 80.6 |  |  | 4.08 |
| 121 | 13629 |  |  | 81.2 |  |  | 3.93 |
| 122 | 14583 |  |  | 87.5 |  | L | 2.49 |
| 123 | 12981 |  |  | 77 |  |  | 5.01 |
| 124 | 14130 |  |  | 84.5 |  |  | 3.15 |
| 125 | 14184 |  |  | 84.9 |  |  | 3.06 |
| 126 | 13890 |  |  | 82.9 |  |  | 3.52 |
| 127 | 13528 |  |  | 80.6 |  |  | 4.08 |
| 128 | 13017 |  |  | 77.2 |  |  | 4.96 |
| 129 | 12942 |  |  | 76.7 |  |  | 5.09 |
| 130 | 15362 |  |  | 92.6 |  | L | 1.43 |
| 131 | 14457 |  |  | 86.7 |  | L | 2.66 |
| 132 | 13638 |  |  | 81.3 |  |  | 3.91 |
| 133 | 13336 |  |  | 79.3 |  |  | 4.41 |
| 134 | 13488 |  |  | 80.3 |  |  | 4.16 |
| 135 | 14636 |  |  | 87.8 |  | L | 2.42 |
| 136 | 14008 |  |  | 83.7 |  |  | 3.34 |
| 137 | 14459 |  |  | 86.7 |  | L | 2.66 |
| 138 | 13350 |  |  | 79.4 |  |  | 4.38 |
| 139 | 12957 |  |  | 76.8 |  |  | 5.06 |
| 140 | 14216 |  |  | 85.1 |  |  | 3.02 |
| 141 | 13517 |  |  | 80.5 |  |  | 4.11 |
| 142 | 13348 |  |  | 79.4 |  |  | 4.38 |
| 143 | 13687 |  |  | 81.6 |  |  | 3.84 |
| 144 | 13515 |  |  | 80.5 |  |  | 4.11 |
| 145 | 15295 |  |  | 92.2 |  | L | 1.51 |
| 146 | 13464 |  |  | 80.1 |  |  | 4.21 |
| 147 | 13718 |  |  | 81.8 |  |  | 3.79 |
| 148 | 14197 |  |  | 85 |  |  | 3.04 |
| 149 | 14135 |  |  | 84.5 |  |  | 3.15 |
| 150 | 14695 |  |  | 88.2 |  | L | 2.34 |
| 151 | 13269 |  |  | 78.9 |  |  | 4.51 |
| 152 | 14842 |  |  | 89.2 |  | L | 2.12 |
| 153 | 15596 |  |  | 94.1 |  | L | 1.14 |
| 154 | 14750 |  |  | 88.6 |  | L | 2.25 |
| 155 | 15275 |  |  | 92 |  | L | 1.55 |
| 156 | 14153 |  |  | 84.7 |  |  | 3.11 |
| 157 | 14459 |  |  | 86.7 |  | L | 2.66 |
| 158 | 14957 |  |  | 89.9 |  | L | 1.98 |
| 159 | 14699 |  |  | 88.2 |  | L | 2.34 |
| 160 | 13049 |  |  | 77.4 |  |  | 4.9 |
| 161 | 14855 |  |  | 89.3 |  | L | 2.1 |
| 162 | 14640 |  |  | 87.9 |  | L | 2.4 |
