# Supplementary material for: AANAT transgenic sheep generated via OPS vitrified-microinjected pronuclear embryos and reproduction efficiency of the transgenic offspring
Source: PeerJ. 2018 Aug 8;6:e5420. doi: 10.7717/peerj.5420 (PMC6087419; doi:10.7717/peerj.5420)
Supplement: Supplemental Information 1 [file peerj-06-5420-s001.zip › Raw data/Detection of MT and hormone/Detection of Gnrh.docx]

| data | 20171020 | | | | | | | | | |
| --- | --- | --- | --- | --- | --- | --- | --- | --- | --- | --- |
| object | Detection of Gnrh | | | | | | | | | |
| materials | PHOENIX PHARMACEUTICALS,INC | | | | | | | | | |
| method | RIA | | | | | | | | | |
| results |  | | | | | | | | | |
|  | CPM1 | CPM2 | Mean CPM | B/B_0_% | log[concentration] | Logit | Self-inspection concentration | |  |  |
| BG | 0 | 0 | 0 |  |  |  |  |  | |  |
| T | 6757 | 6757 | 6757 |  |  |  |  |  | |  |
| NSB | 83 | 83 | 82.95 |  |  |  |  | NSB/T%= | | 1.228 |
| 0 | 1076 | 1076 | 1076.04 | 100.00 |  |  |  | B_0_/T%= | | 14.697 |
| 2.5 | 948 | 948 | 947.52 | 87.06 | 0.398 | 1.906 | 2.67 | A= | | 2.781 |
| 5 | 874 | 874 | 874.44 | 79.70 | 0.699 | 1.368 | 4.89 | B= | | -2.051 |
| 10 | 750 | 750 | 749.7 | 67.14 | 1.000 | 0.714 | 10.17 | R= | | -0.995 |
| 20 | 668 | 668 | 667.8 | 58.89 | 1.301 | 0.360 | 15.15 | ED75= | | 6.607 |
| 40 | 391 | 391 | 390.6 | 30.98 | 1.602 | -0.801 | 55.74 | ED50= | | 22.678 |
| 80 | 340 | 340 | 340.2 | 25.90 | 1.903 | -1.051 | 73.78 | ED25= | | 77.838 |
| 160 | 246 | 246 | 245.7 | 16.39 | 2.204 | -1.630 | 141.27 |  | |  |
| 320 | 163 | 163 | 162.54 | 8.01 | 2.505 | -2.440 | 351.01 |  | |  |
| \|  \| \| --- \| |  |  |  |  |  |  |  |  | |  |
|  |  |  |  |  |  |  |  |  | |  |
|  |  |  |  |  |  |  |  |  | |  |
|  |  |  |  |  |  |  |  |  | |  |
|  |  |  |  |  |  |  |  |  | |  |
|  |  |  |  |  |  |  |  |  | |  |
|  |  |  |  |  |  |  |  |  | |  |
|  |  |  |  |  |  |  |  |  | |  |
|  |  |  |  |  |  |  |  |  | |  |
|  |  |  |  |  |  |  |  |  | |  |
|  |  |  |  |  |  |  |  |  | |  |
|  |  |  |  |  |  |  |  |  | |  |
|  |  |  |  |  |  |  |  |  | |  |
|  |  |  |  |  |  |  |  |  | |  |
|  |  |  |  |  |  |  |  |  | |  |
|  |  |  |  |  |  |  |  |  | |  |
|  |  |  |  |  |  |  |  |  | |  |
| sample | CPM1 | CPM2 | Mean CPM | B/B_0_% | Logit |  | Concentration(pg/ml) |  | |  |
| QC | 396 |  | 396 | 31.52 | -0.776 |  | 54.18 |  | |  |
| 1 | 563 |  | 563 | 48.34 | -0.066 |  | 24.44 |  | |  |
| 2 | 693 |  | 693 | 61.43 | 0.465 |  | 13.45 |  | |  |
| 3 | 735 |  | 735 | 65.66 | 0.648 |  | 10.96 |  | |  |
| 4 | 684 |  | 684 | 60.52 | 0.427 |  | 14.04 |  | |  |
| 5 | 895 |  | 895 | 81.77 | 1.501 |  | 4.21 |  | |  |
| 6 | 641 |  | 641 | 56.19 | 0.249 |  | 17.15 |  | |  |
| 7 | 553 |  | 553 | 47.33 | -0.107 |  | 25.57 |  | |  |
| 8 | 593 |  | 593 | 51.36 | 0.054 |  | 21.34 |  | |  |
| 9 | 732 |  | 732 | 65.36 | 0.635 |  | 11.12 |  | |  |
| 10 | 634 |  | 634 | 55.49 | 0.220 |  | 17.71 |  | |  |
| 11 | 560 |  | 560 | 48.04 | -0.079 |  | 24.77 |  | |  |
| 12 | 643 |  | 643 | 56.39 | 0.257 |  | 16.99 |  | |  |
| 13 | 616 |  | 616 | 53.68 | 0.147 |  | 19.22 |  | |  |
| 14 | 603 |  | 603 | 52.37 | 0.095 |  | 20.39 |  | |  |
| 15 | 650 |  | 650 | 57.10 | 0.286 |  | 16.45 |  | |  |
| 16 | 614 |  | 614 | 53.47 | 0.139 |  | 19.40 |  | |  |
| 17 | 702 |  | 702 | 62.34 | 0.504 |  | 12.88 |  | |  |
| 18 | 622 |  | 622 | 54.28 | 0.172 |  | 18.70 |  | |  |
| 19 | 605 |  | 605 | 52.57 | 0.103 |  | 20.21 |  | |  |
| 20 | 605 |  | 605 | 52.57 | 0.103 |  | 20.21 |  | |  |
| 21 | 582 |  | 582 | 50.25 | 0.010 |  | 22.42 |  | |  |
| 22 | 728 |  | 728 | 64.95 | 0.617 |  | 11.35 |  | |  |
| 23 | 567 |  | 567 | 48.74 | -0.050 |  | 24.00 |  | |  |
| 24 | 668 |  | 668 | 58.91 | 0.360 |  | 15.13 |  | |  |
| 25 | 686 |  | 686 | 60.72 | 0.436 |  | 13.91 |  | |  |
| 26 | 621 |  | 621 | 54.18 | 0.168 |  | 18.79 |  | |  |
| 27 | 779 |  | 779 | 70.09 | 0.852 |  | 8.72 |  | |  |
| 28 | 654 |  | 654 | 57.50 | 0.302 |  | 16.15 |  | |  |
| 29 | 636 |  | 636 | 55.69 | 0.229 |  | 17.55 |  | |  |
| 30 | 686 |  | 686 | 60.72 | 0.436 |  | 13.91 |  | |  |
| 31 | 601 |  | 601 | 52.17 | 0.087 |  | 20.58 |  | |  |
| 32 | 653 |  | 653 | 57.40 | 0.298 |  | 16.23 |  | |  |
| 33 | 564 |  | 564 | 48.44 | -0.062 |  | 24.33 |  | |  |
| 34 | 479 |  | 479 | 39.88 | -0.410 |  | 35.95 |  | |  |
| 35 | 626 |  | 626 | 54.68 | 0.188 |  | 18.37 |  | |  |
| 36 | 703 |  | 703 | 62.44 | 0.508 |  | 12.82 |  | |  |
| 37 | 513 |  | 513 | 43.30 | -0.269 |  | 30.69 |  | |  |
| 38 | 576 |  | 576 | 49.65 | -0.014 |  | 23.04 |  | |  |
| 39 | 619 |  | 619 | 53.98 | 0.159 |  | 18.96 |  | |  |
| 40 | 602 |  | 602 | 52.27 | 0.091 |  | 20.48 |  | |  |
| 41 | 650 |  | 650 | 57.10 | 0.286 |  | 16.45 |  | |  |
| 42 | 594 |  | 594 | 51.46 | 0.058 |  | 21.24 |  | |  |
| 43 | 667 |  | 667 | 58.81 | 0.356 |  | 15.20 |  | |  |
| 44 | 648 |  | 648 | 56.90 | 0.278 |  | 16.61 |  | |  |
| 45 | 581 |  | 581 | 50.15 | 0.006 |  | 22.52 |  | |  |
| 46 | 461 |  | 461 | 38.07 | -0.487 |  | 39.16 |  | |  |
| 47 | 472 |  | 472 | 39.18 | -0.440 |  | 37.16 |  | |  |
| 48 | 571 |  | 571 | 49.14 | -0.034 |  | 23.57 |  | |  |
| 49 | 817 |  | 817 | 73.92 | 1.042 |  | 7.04 |  | |  |
| 50 | 614 |  | 614 | 53.47 | 0.139 |  | 19.40 |  | |  |
| 51 | 645 |  | 645 | 56.60 | 0.265 |  | 16.84 |  | |  |
| 52 | 665 |  | 665 | 58.61 | 0.348 |  | 15.35 |  | |  |
| 53 | 588 |  | 588 | 50.86 | 0.034 |  | 21.82 |  | |  |
| 54 | 632 |  | 632 | 55.29 | 0.212 |  | 17.87 |  | |  |
| 55 | 619 |  | 619 | 53.98 | 0.159 |  | 18.96 |  | |  |
| 56 | 744 |  | 744 | 66.56 | 0.689 |  | 10.47 |  | |  |
| 57 | 601 |  | 601 | 52.17 | 0.087 |  | 20.58 |  | |  |
| 58 | 627 |  | 627 | 54.78 | 0.192 |  | 18.28 |  | |  |
| 59 | 629 |  | 629 | 54.98 | 0.200 |  | 18.12 |  | |  |
| 60 | 562 |  | 562 | 48.24 | -0.070 |  | 24.55 |  | |  |
| 61 | 781 |  | 781 | 70.29 | 0.861 |  | 8.63 |  | |  |
| 62 | 643 |  | 643 | 56.39 | 0.257 |  | 16.99 |  | |  |
| 63 | 620 |  | 620 | 54.08 | 0.164 |  | 18.88 |  | |  |
| 64 | 661 |  | 661 | 58.21 | 0.331 |  | 15.64 |  | |  |
| 65 | 630 |  | 630 | 55.09 | 0.204 |  | 18.03 |  | |  |
| 66 | 643 |  | 643 | 56.39 | 0.257 |  | 16.99 |  | |  |
| 67 | 669 |  | 669 | 59.01 | 0.364 |  | 15.06 |  | |  |
| 68 | 620 |  | 620 | 54.08 | 0.164 |  | 18.88 |  | |  |
| 69 | 770 |  | 770 | 69.18 | 0.809 |  | 9.15 |  | |  |
| 70 | 715 |  | 715 | 63.64 | 0.560 |  | 12.10 |  | |  |
| 71 | 609 |  | 609 | 52.97 | 0.119 |  | 19.84 |  | |  |
| 72 | 673 |  | 673 | 59.42 | 0.381 |  | 14.78 |  | |  |
| 73 | 599 |  | 599 | 51.96 | 0.079 |  | 20.76 |  | |  |
| 74 | 711 |  | 711 | 63.24 | 0.543 |  | 12.33 |  | |  |
| 75 | 681 |  | 681 | 60.22 | 0.415 |  | 14.24 |  | |  |
| 76 | 693 |  | 693 | 61.43 | 0.465 |  | 13.45 |  | |  |
| 77 | 630 |  | 630 | 55.09 | 0.204 |  | 18.03 |  | |  |
| 78 | 689 |  | 689 | 61.03 | 0.448 |  | 13.71 |  | |  |
| 79 | 608 |  | 608 | 52.87 | 0.115 |  | 19.93 |  | |  |
| 80 | 778 |  | 778 | 69.99 | 0.847 |  | 8.77 |  | |  |
| 81 | 709 |  | 709 | 63.04 | 0.534 |  | 12.45 |  | |  |
| 82 | 973 |  | 973 | 89.62 | 2.156 |  | 2.02 |  | |  |
| 83 | 691 |  | 691 | 61.23 | 0.457 |  | 13.58 |  | |  |
| 84 | 544 |  | 544 | 46.43 | -0.143 |  | 26.63 |  | |  |
| 85 | 568 |  | 568 | 48.84 | -0.046 |  | 23.89 |  | |  |
| 86 | 508 |  | 508 | 42.80 | -0.290 |  | 31.40 |  | |  |
| 87 | 592 |  | 592 | 51.26 | 0.050 |  | 21.43 |  | |  |
| 88 | 627 |  | 627 | 54.78 | 0.192 |  | 18.28 |  | |  |
| 89 | 597 |  | 597 | 51.76 | 0.071 |  | 20.95 |  | |  |
| 90 | 530 |  | 530 | 45.02 | -0.200 |  | 28.39 |  | |  |
| 91 | 604 |  | 604 | 52.47 | 0.099 |  | 20.30 |  | |  |
| 92 | 742 |  | 742 | 66.36 | 0.680 |  | 10.58 |  | |  |
| 93 | 696 |  | 696 | 61.73 | 0.478 |  | 13.26 |  | |  |
| 94 | 688 |  | 688 | 60.93 | 0.444 |  | 13.77 |  | |  |
| 95 | 746 |  | 746 | 66.77 | 0.698 |  | 10.36 |  | |  |
| 96 | 602 |  | 602 | 52.27 | 0.091 |  | 20.48 |  | |  |
| 97 | 574 |  | 574 | 49.45 | -0.022 |  | 23.25 |  | |  |
| 98 | 597 |  | 597 | 51.76 | 0.071 |  | 20.95 |  | |  |
| 99 | 632 |  | 632 | 55.29 | 0.212 |  | 17.87 |  | |  |
| 100 | 646 |  | 646 | 56.70 | 0.269 |  | 16.76 |  | |  |
| 101 | 669 |  | 669 | 59.01 | 0.364 |  | 15.06 |  | |  |
| 102 | 700 |  | 700 | 62.13 | 0.495 |  | 13.01 |  | |  |
| 103 | 635 |  | 635 | 55.59 | 0.225 |  | 17.63 |  | |  |
| 104 | 632 |  | 632 | 55.29 | 0.212 |  | 17.87 |  | |  |
| 105 | 681 |  | 681 | 60.22 | 0.415 |  | 14.24 |  | |  |
| 106 | 636 |  | 636 | 55.69 | 0.229 |  | 17.55 |  | |  |
| 107 | 580 |  | 580 | 50.05 | 0.002 |  | 22.63 |  | |  |
| 108 | 629 |  | 629 | 54.98 | 0.200 |  | 18.12 |  | |  |
| 109 | 580 |  | 580 | 50.05 | 0.002 |  | 22.63 |  | |  |
| 110 | 559 |  | 559 | 47.94 | -0.083 |  | 24.88 |  | |  |
| 111 | 614 |  | 614 | 53.47 | 0.139 |  | 19.40 |  | |  |
| 112 | 576 |  | 576 | 49.65 | -0.014 |  | 23.04 |  | |  |
| 113 | 686 |  | 686 | 60.72 | 0.436 |  | 13.91 |  | |  |
