# Supplementary material for: AANAT transgenic sheep generated via OPS vitrified-microinjected pronuclear embryos and reproduction efficiency of the transgenic offspring
Source: PeerJ. 2018 Aug 8;6:e5420. doi: 10.7717/peerj.5420 (PMC6087419; doi:10.7717/peerj.5420)
Supplement: Supplemental Information 1 [file peerj-06-5420-s001.zip › Raw data/Detection of MT and hormone/Detection of LH.docx]

|  | **research data report of Beijing north institute of biotechnology co. LTD.** | | | | | | |
| --- | --- | --- | --- | --- | --- | --- | --- |
| data | 20171028 | | | | | | |
| Applicant Name | China Agricultural University | | | | | | |
| consignor | Xiuzhi Tian | | | contact information |  | | |
|  |  |  |  | E-MAIL |  | | |
| test item | LH | | | | | | |
| Reagent batches | 20171020 | | | | | | |
| The reagent manufacturer | Beijing north institute of biotechnology co. LTD. | | | | | | |
| detection method | The standard products and samples were labeled with the antibody and incubated at 4℃ for 20hr, and then added separation agent and centrifuged for 15min at 3800rpm | | | | | | |
| instrument | Xi 'an nuclear instrument factory xh6080 radio-immunity analyzer | | | | | | |
| results |  |  |  |  |  |  |  |
| T | 32157 | 32157 |  | 0 |  |  |  |
| N | 1626 | 1626 | 1626 | 5.1 |  | r= | -0.99441 |
| 0 | 18521 | 18521 | 16895 | 52.5 |  | a= | 3.22 |
| 5 | 16240 | 16240 |  | 86.5 |  | b= | -2.14 |
| 10 | 13747 | 13747 |  | 71.7 |  |  |  |
| 25 | 10658 | 10658 |  | 53.5 |  | ed75= | 9.8 |
| 50 | 8365 | 8365 |  | 39.9 |  | ed50= | 31.96 |
| 100 | 6629 | 6629 |  | 29.6 |  | ed25= | 104.23 |
| 200 | 4025 | 4025 |  | 14.2 |  |  |  |
| ＱＣL： | 13419 |  |  | 69.8 |  |  | 12.98 |
| ＱＣH： | 5902 |  |  | 25.3 |  |  | 102.46 |
| numerical order |  |  |  |  |  |  | measurement value (mIU/ml) |
| 1 | 14601 |  |  | 76.8 |  |  | 8.82 |
| 2 | 14709 |  |  | 77.4 |  |  | 8.5 |
| 3 | 13783 |  |  | 72 |  |  | 11.57 |
| 4 | 16782 |  |  | 89.7 |  |  | 3.11 |
| 5 | 16295 |  |  | 86.8 |  |  | 4.21 |
| 6 | 14815 |  |  | 78.1 |  |  | 8.14 |
| 7 | 14831 |  |  | 78.2 |  |  | 8.09 |
| 8 | 15601 |  |  | 82.7 |  |  | 5.94 |
| 9 | 16719 |  |  | 89.3 |  |  | 3.26 |
| 10 | 16124 |  |  | 85.8 |  |  | 4.62 |
| 11 | 15130 |  |  | 79.9 |  |  | 7.24 |
| 12 | 15222 |  |  | 80.5 |  |  | 6.95 |
| 13 | 15073 |  |  | 79.6 |  |  | 7.39 |
| 14 | 15910 |  |  | 84.5 |  |  | 5.16 |
| 15 | 14901 |  |  | 78.6 |  |  | 7.88 |
| 16 | 16192 |  |  | 86.2 |  |  | 4.45 |
| 17 | 16155 |  |  | 86 |  |  | 4.53 |
| 18 | 16325 |  |  | 87 |  |  | 4.13 |
| 19 | 15476 |  |  | 82 |  |  | 6.25 |
| 20 | 15841 |  |  | 84.1 |  |  | 5.33 |
| 21 | 12967 |  |  | 67.1 |  |  | 14.85 |
| 22 | 15931 |  |  | 84.7 |  |  | 5.07 |
| 23 | 14816 |  |  | 78.1 |  |  | 8.14 |
| 24 | 15322 |  |  | 81.1 |  |  | 6.67 |
| 25 | 14198 |  |  | 74.4 |  |  | 10.14 |
| 26 | 14640 |  |  | 77 |  |  | 8.71 |
| 27 | 16045 |  |  | 85.3 |  |  | 4.82 |
| 28 | 14592 |  |  | 76.7 |  |  | 8.87 |
| 29 | 15588 |  |  | 82.6 |  |  | 5.98 |
| 30 | 16228 |  |  | 86.4 |  |  | 4.37 |
| 31 | 14916 |  |  | 78.7 |  |  | 7.83 |
| 32 | 15297 |  |  | 80.9 |  |  | 6.76 |
| 33 | 15288 |  |  | 80.9 |  |  | 6.76 |
| 34 | 15930 |  |  | 84.7 |  |  | 5.07 |
| 35 | 16195 |  |  | 86.2 |  |  | 4.45 |
| 36 | 16613 |  |  | 88.7 |  |  | 3.48 |
| 37 | 14588 |  |  | 76.7 |  |  | 8.87 |
| 38 | 14763 |  |  | 77.8 |  |  | 8.29 |
| 39 | 14332 |  |  | 75.2 |  |  | 9.69 |
| 40 | 16926 |  |  | 90.6 |  |  | 2.79 |
| 41 | 15723 |  |  | 83.4 |  |  | 5.63 |
| 42 | 14792 |  |  | 77.9 |  |  | 8.24 |
| 43 | 14732 |  |  | 77.6 |  |  | 8.4 |
| 44 | 16230 |  |  | 86.4 |  |  | 4.37 |
| 45 | 16747 |  |  | 89.5 |  |  | 3.19 |
| 46 | 15954 |  |  | 84.8 |  |  | 5.03 |
| 47 | 15456 |  |  | 81.9 |  |  | 6.3 |
| 48 | 15314 |  |  | 81 |  |  | 6.72 |
| 49 | 14794 |  |  | 77.9 |  |  | 8.24 |
| 50 | 16346 |  |  | 87.1 |  |  | 4.1 |
| 51 | 14729 |  |  | 77.6 |  |  | 8.4 |
| 52 | 15826 |  |  | 84 |  |  | 5.37 |
| 53 | 16145 |  |  | 85.9 |  |  | 4.57 |
| 54 | 16667 |  |  | 89 |  |  | 3.37 |
| 55 | 15847 |  |  | 84.2 |  |  | 5.28 |
| 56 | 16138 |  |  | 85.9 |  |  | 4.57 |
| 57 | 14390 |  |  | 75.5 |  |  | 9.52 |
| 58 | 15902 |  |  | 84.5 |  |  | 5.16 |
| 59 | 15509 |  |  | 82.2 |  |  | 6.16 |
| 60 | 15913 |  |  | 84.6 |  |  | 5.11 |
| 61 | 14919 |  |  | 78.7 |  |  | 7.83 |
| 62 | 16035 |  |  | 85.3 |  |  | 4.82 |
| 63 | 16564 |  |  | 88.4 |  |  | 3.6 |
| 64 | 16169 |  |  | 86.1 |  |  | 4.49 |
| 65 | 16493 |  |  | 88 |  |  | 3.75 |
| 66 | 16374 |  |  | 87.3 |  |  | 4.02 |
| 67 | 15025 |  |  | 79.3 |  |  | 7.54 |
| 68 | 16061 |  |  | 85.4 |  |  | 4.78 |
| 69 | 15454 |  |  | 81.8 |  |  | 6.35 |
| 70 | 16121 |  |  | 85.8 |  |  | 4.62 |
| 71 | 16607 |  |  | 88.7 |  |  | 3.48 |
| 72 | 16353 |  |  | 87.2 |  |  | 4.06 |
| 73 | 15650 |  |  | 83 |  |  | 5.8 |
| 74 | 15858 |  |  | 84.2 |  |  | 5.28 |
| 75 | 14354 |  |  | 75.3 |  |  | 9.63 |
| 76 | 17110 |  |  | 91.6 |  |  | 2.45 |
| 77 | 15797 |  |  | 83.9 |  |  | 5.41 |
| 78 | 15091 |  |  | 79.7 |  |  | 7.34 |
| 79 | 15308 |  |  | 81 |  |  | 6.72 |
| 80 | 15774 |  |  | 83.7 |  |  | 5.5 |
| 81 | 15130 |  |  | 79.9 |  |  | 7.24 |
| 82 | 15751 |  |  | 83.6 |  |  | 5.54 |
| 83 | 15893 |  |  | 84.4 |  |  | 5.2 |
| 84 | 15221 |  |  | 80.5 |  |  | 6.95 |
| 85 | 15387 |  |  | 81.5 |  |  | 6.48 |
| 86 | 16483 |  |  | 87.9 |  |  | 3.79 |
| 87 | 15352 |  |  | 81.2 |  |  | 6.62 |
| 88 | 14920 |  |  | 78.7 |  |  | 7.83 |
| 89 | 16349 |  |  | 87.1 |  |  | 4.1 |
| 90 | 16770 |  |  | 89.6 |  |  | 3.15 |
| 91 | 15140 |  |  | 80 |  |  | 7.19 |
| 92 | 15073 |  |  | 79.6 |  |  | 7.39 |
| 93 | 14694 |  |  | 77.3 |  |  | 8.55 |
| 94 | 16710 |  |  | 89.3 |  |  | 3.26 |
| 95 | 16600 |  |  | 88.6 |  |  | 3.52 |
| 96 | 15157 |  |  | 80.1 |  |  | 7.14 |
| 97 | 14806 |  |  | 78 |  |  | 8.19 |
| 98 | 14494 |  |  | 76.2 |  |  | 9.14 |
| 99 | 16328 |  |  | 87 |  |  | 4.13 |
| 100 | 15746 |  |  | 83.6 |  |  | 5.54 |
| 101 | 14411 |  |  | 75.7 |  |  | 9.41 |
| 102 | 15183 |  |  | 80.2 |  |  | 7.1 |
| 103 | 14662 |  |  | 77.2 |  |  | 8.61 |
| 104 | 16203 |  |  | 86.3 |  |  | 4.41 |
| 105 | 15180 |  |  | 80.2 |  |  | 7.1 |
| 106 | 15666 |  |  | 83.1 |  |  | 5.76 |
| 107 | 16332 |  |  | 87 |  |  | 4.13 |
| 108 | 15858 |  |  | 84.2 |  |  | 5.28 |
| 109 | 14719 |  |  | 77.5 |  |  | 8.45 |
| 110 | 16450 |  |  | 87.7 |  |  | 3.86 |
| 111 | 14348 |  |  | 75.3 |  |  | 9.63 |
| 112 | 15353 |  |  | 81.2 |  |  | 6.62 |
| 113 | 14783 |  |  | 77.9 |  |  | 8.24 |
| 114 | 14687 |  |  | 77.3 |  |  | 8.55 |
| 115 | 15224 |  |  | 80.5 |  |  | 6.95 |
| 116 | 15339 |  |  | 81.2 |  |  | 6.62 |
| 117 | 15595 |  |  | 82.7 |  |  | 5.94 |
| 118 | 14825 |  |  | 78.1 |  |  | 8.14 |
| 119 | 15818 |  |  | 84 |  |  | 5.37 |
| 120 | 15359 |  |  | 81.3 |  |  | 6.58 |
| 121 | 14929 |  |  | 78.7 |  |  | 7.83 |
| 122 | 15621 |  |  | 82.8 |  |  | 5.89 |
| 123 | 15316 |  |  | 81 |  |  | 6.72 |
| 124 | 16101 |  |  | 85.7 |  |  | 4.66 |
| 125 | 16210 |  |  | 86.3 |  |  | 4.41 |
| 126 | 16456 |  |  | 87.8 |  |  | 3.82 |
| 127 | 15179 |  |  | 80.2 |  |  | 7.1 |
| 128 | 15326 |  |  | 81.1 |  |  | 6.67 |
| 129 | 13901 |  |  | 72.7 |  |  | 11.14 |
| 130 | 16508 |  |  | 88.1 |  |  | 3.71 |
| 131 | 15597 |  |  | 82.7 |  |  | 5.94 |
| 132 | 14763 |  |  | 77.8 |  |  | 8.29 |
| 133 | 14867 |  |  | 78.4 |  |  | 7.99 |
| 134 | 15813 |  |  | 84 |  |  | 5.37 |
| 135 | 16981 |  |  | 90.9 |  | L | 2.69 |
| 136 | 15698 |  |  | 83.3 |  |  | 5.67 |
| 137 | 15283 |  |  | 80.8 |  |  | 6.81 |
| 138 | 14863 |  |  | 78.3 |  |  | 8.04 |
| 139 | 14456 |  |  | 75.9 |  |  | 9.3 |
| 140 | 14894 |  |  | 78.5 |  |  | 7.93 |
| 141 | 14547 |  |  | 76.5 |  |  | 8.98 |
| 142 | 15284 |  |  | 80.8 |  |  | 6.81 |
| 143 | 16375 |  |  | 87.3 |  |  | 4.02 |
| 144 | 16214 |  |  | 86.3 |  |  | 4.41 |
| 145 | 15409 |  |  | 81.6 |  |  | 6.44 |
| 146 | 14687 |  |  | 77.3 |  |  | 8.55 |
| 147 | 14685 |  |  | 77.3 |  |  | 8.55 |
| 148 | 15736 |  |  | 83.5 |  |  | 5.58 |
| 149 | 15096 |  |  | 79.7 |  |  | 7.34 |
| 150 | 15697 |  |  | 83.3 |  |  | 5.67 |
| 151 | 13816 |  |  | 72.2 |  |  | 11.45 |
| 152 | 15157 |  |  | 80.1 |  |  | 7.14 |
| 153 | 15751 |  |  | 83.6 |  |  | 5.54 |
| 154 | 15299 |  |  | 80.9 |  |  | 6.76 |
| 155 | 15663 |  |  | 83.1 |  |  | 5.76 |
| 156 | 14151 |  |  | 74.1 |  |  | 10.32 |
| 157 | 14919 |  |  | 78.7 |  |  | 7.83 |
| 158 | 14185 |  |  | 74.3 |  |  | 10.2 |
| 159 | 15510 |  |  | 82.2 |  |  | 6.16 |
| 160 | 15105 |  |  | 79.8 |  |  | 7.29 |
| 161 | 16403 |  |  | 87.5 |  |  | 3.94 |
| 162 | 15791 |  |  | 83.8 |  |  | 5.45 |
