# Supplementary material for: AANAT transgenic sheep generated via OPS vitrified-microinjected pronuclear embryos and reproduction efficiency of the transgenic offspring
Source: PeerJ. 2018 Aug 8;6:e5420. doi: 10.7717/peerj.5420 (PMC6087419; doi:10.7717/peerj.5420)
Supplement: Supplemental Information 1 [file peerj-06-5420-s001.zip › Raw data/Detection of MT and hormone/Detection of Melatonin.docx]

| data | 201701028 | | | | | | | | |
| --- | --- | --- | --- | --- | --- | --- | --- | --- | --- |
| object | Detection of melatonin | | | | | | | | |
| material | LDN Melatonin Direct RIA | | | | | | | | |
| method | RIA | | | | | | | | |
| result |  | | | | | | | | |
|  | CPM1 | CPM2 | Mean CPM | B/B_0_% | log[concentration] | Logit | Self-inspection concentration |  |  |
| BG | 0 | 0 | 0 |  |  |  |  |  |  |
| T | 26747 | 26747 | 26747 |  |  |  |  |  |  |
| NSB | 620 | 620 | 620 |  |  |  |  | NSB/T%= | 2.3180 |
| 0 | 3922 | 3922 | 3922 | 100.00 |  |  |  | B_0_/T%= | 12.35 |
| 30 | 3765 | 3765 | 3765 | 95.25 | 1.477 | 2.997 | 23.89 | A= | 5.24882 |
| 100 | 3396 | 3396 | 3396 | 84.07 | 2.000 | 1.663 | 156.61 | B= | -1.63354 |
| 300 | 3258 | 3258 | 3258 | 79.89 | 2.477 | 1.379 | 233.71 | R= | -0.99324 |
| 1000 | 2528 | 2528 | 2528 | 57.78 | 3.000 | 0.314 | 1049.55 | ED75= | 347.228 |
| 3000 | 1911 | 1911 | 1911 | 39.10 | 3.477 | -0.443 | 3051.19 | ED50= | 1633.607 |
| 10000 | 1350 | 1350 | 1350 | 22.11 | 4.000 | -1.259 | 9640.65 | ED25= | 7685.652 |
| \|  \| \| --- \| |  |  |  |  |  |  |  |  |  |
|  |  |  |  |  |  |  |  |  |  |
|  |  |  |  |  |  |  |  |  |  |
|  |  |  |  |  |  |  |  |  |  |
|  |  |  |  |  |  |  |  |  |  |
|  |  |  |  |  |  |  |  |  |  |
|  |  |  |  |  |  |  |  |  |  |
|  |  |  |  |  |  |  |  |  |  |
|  |  |  |  |  |  |  |  |  |  |
|  |  |  |  |  |  |  |  |  |  |
|  |  |  |  |  |  |  |  |  |  |
|  |  |  |  |  |  |  |  |  |  |
|  |  |  |  |  |  |  |  |  |  |
|  |  |  |  |  |  |  |  |  |  |
|  |  |  |  |  |  |  |  |  |  |
|  |  |  |  |  |  |  |  |  |  |
|  |  |  |  |  |  |  |  |  |  |
| sample | CPM1 | CPM2 | Mean CPM | B/B_0_% | Logit |  | Concentration(pg/ml) |  |  |
| L | 3414 |  | 3414 | 84.62 | 1.705 |  | 147.76 |  |  |
| M |  |  |  |  |  |  |  |  |  |
| H | 3030 |  | 3030 | 72.99 | 0.994 |  | 402.45 |  |  |
| 1 | 3537 |  | 3537 | 88.34 | 2.025 |  | 94.07 |  |  |
| 2 | 2618 |  | 2618 | 60.51 | 0.427 |  | 895.22 |  |  |
| 3 | 2020 |  | 2020 | 42.40 | -0.306 |  | 2516.15 |  |  |
| 4 | 2567 |  | 2567 | 58.96 | 0.362 |  | 980.04 |  |  |
| 5 | 2977 |  | 2977 | 71.38 | 0.914 |  | 450.46 |  |  |
| 6 | 3296 |  | 3296 | 81.04 | 1.453 |  | 210.78 |  |  |
| 7 | 2266 |  | 2266 | 49.85 | -0.006 |  | 1647.61 |  |  |
| 8 | 3285 |  | 3285 | 80.71 | 1.431 |  | 217.28 |  |  |
| 9 | 2148 |  | 2148 | 46.27 | -0.149 |  | 2016.18 |  |  |
| 10 | 2085 |  | 2085 | 44.37 | -0.226 |  | 2247.33 |  |  |
| 11 | 3206 |  | 3206 | 78.32 | 1.284 |  | 267.31 |  |  |
| 12 | 3340 |  | 3340 | 82.37 | 1.542 |  | 185.88 |  |  |
| 13 | 2528 |  | 2528 | 57.78 | 0.314 |  | 1049.55 |  |  |
| 14 | 2457 |  | 2457 | 55.63 | 0.226 |  | 1187.48 |  |  |
| 15 | 3219 |  | 3219 | 78.71 | 1.308 |  | 258.66 |  |  |
| 16 | 2145 |  | 2145 | 46.18 | -0.153 |  | 2026.60 |  |  |
| 17 | 2201 |  | 2201 | 47.88 | -0.085 |  | 1841.15 |  |  |
| 18 | 2148 |  | 2148 | 46.27 | -0.149 |  | 2016.18 |  |  |
| 19 | 2756 |  | 2756 | 64.69 | 0.605 |  | 695.94 |  |  |
| 20 | 3226 |  | 3226 | 78.92 | 1.320 |  | 254.07 |  |  |
| 21 | 2169 |  | 2169 | 46.91 | -0.124 |  | 1944.84 |  |  |
| 22 | 3520 |  | 3520 | 87.83 | 1.976 |  | 100.81 |  |  |
| 23 | 2275 |  | 2275 | 50.12 | 0.005 |  | 1622.49 |  |  |
| 24 | 2120 |  | 2120 | 45.43 | -0.183 |  | 2115.62 |  |  |
| 25 | 3213 |  | 3213 | 78.53 | 1.297 |  | 262.63 |  |  |
| 26 | 2390 |  | 2390 | 53.60 | 0.144 |  | 1332.75 |  |  |
| 27 | 2382 |  | 2382 | 53.36 | 0.135 |  | 1351.17 |  |  |
| 28 | 2354 |  | 2354 | 52.51 | 0.101 |  | 1417.57 |  |  |
| 29 | 2627 |  | 2627 | 60.78 | 0.438 |  | 880.93 |  |  |
| 30 | 2012 |  | 2012 | 42.16 | -0.316 |  | 2551.61 |  |  |
| 31 | 2274 |  | 2274 | 50.09 | 0.004 |  | 1625.26 |  |  |
| 32 | 2370 |  | 2370 | 53.00 | 0.120 |  | 1379.25 |  |  |
| 33 | 2829 |  | 2829 | 66.90 | 0.704 |  | 605.93 |  |  |
| 34 | 3526 |  | 3526 | 88.01 | 1.993 |  | 98.41 |  |  |
| 35 | 2714 |  | 2714 | 63.42 | 0.550 |  | 752.30 |  |  |
| 36 | 3253 |  | 3253 | 79.74 | 1.370 |  | 236.82 |  |  |
| 37 | 2555 |  | 2555 | 58.60 | 0.347 |  | 1000.98 |  |  |
| 38 | 2277 |  | 2277 | 50.18 | 0.007 |  | 1616.96 |  |  |
| 39 | 2594 |  | 2594 | 59.78 | 0.396 |  | 934.31 |  |  |
| 40 | 3046 |  | 3046 | 73.47 | 1.019 |  | 388.67 |  |  |
| 41 | 2732 |  | 2732 | 63.96 | 0.574 |  | 727.71 |  |  |
| 42 | 2847 |  | 2847 | 67.44 | 0.728 |  | 585.18 |  |  |
| 43 | 3026 |  | 3026 | 72.86 | 0.988 |  | 405.94 |  |  |
| 44 | 2281 |  | 2281 | 50.30 | 0.012 |  | 1605.95 |  |  |
| 45 | 2527 |  | 2527 | 57.75 | 0.313 |  | 1051.39 |  |  |
| 46 | 2651 |  | 2651 | 61.51 | 0.469 |  | 843.74 |  |  |
| 47 | 2590 |  | 2590 | 59.66 | 0.391 |  | 940.97 |  |  |
| 48 | 3568 |  | 3568 | 89.28 | 2.120 |  | 82.34 |  |  |
| 49 | 2627 |  | 2627 | 60.78 | 0.438 |  | 880.93 |  |  |
| 50 | 3241 |  | 3241 | 79.38 | 1.348 |  | 244.40 |  |  |
| 51 | 2803 |  | 2803 | 66.11 | 0.668 |  | 636.88 |  |  |
| 52 | 2441 |  | 2441 | 55.15 | 0.207 |  | 1220.77 |  |  |
| 53 | 3633 |  | 3633 | 91.25 | 2.344 |  | 59.99 |  |  |
| 54 | 2365 |  | 2365 | 52.85 | 0.114 |  | 1391.12 |  |  |
| 55 | 3130 |  | 3130 | 76.01 | 1.153 |  | 321.39 |  |  |
| 56 | 2429 |  | 2429 | 54.78 | 0.192 |  | 1246.29 |  |  |
| 57 | 3154 |  | 3154 | 76.74 | 1.194 |  | 303.65 |  |  |
| 58 | 2523 |  | 2523 | 57.63 | 0.308 |  | 1058.77 |  |  |
| 59 | 2112 |  | 2112 | 45.18 | -0.193 |  | 2144.98 |  |  |
| 60 | 2851 |  | 2851 | 67.57 | 0.734 |  | 580.64 |  |  |
| 61 | 2997 |  | 2997 | 71.99 | 0.944 |  | 431.90 |  |  |
| 62 | 3194 |  | 3194 | 77.95 | 1.263 |  | 275.44 |  |  |
| 63 | 2493 |  | 2493 | 56.72 | 0.271 |  | 1115.62 |  |  |
| 64 | 3331 |  | 3331 | 82.10 | 1.523 |  | 190.84 |  |  |
| 65 | 2901 |  | 2901 | 69.08 | 0.804 |  | 526.10 |  |  |
| 66 | 2373 |  | 2373 | 53.09 | 0.124 |  | 1372.18 |  |  |
| 67 | 3236 |  | 3236 | 79.22 | 1.339 |  | 247.60 |  |  |
| 68 | 3497 |  | 3497 | 87.13 | 1.912 |  | 110.26 |  |  |
| 69 | 2781 |  | 2781 | 65.45 | 0.639 |  | 664.02 |  |  |
| 70 | 2845 |  | 2845 | 67.38 | 0.726 |  | 587.45 |  |  |
| 71 | 2495 |  | 2495 | 56.78 | 0.273 |  | 1111.75 |  |  |
| 72 | 2662 |  | 2662 | 61.84 | 0.483 |  | 827.15 |  |  |
| 73 | 2916 |  | 2916 | 69.53 | 0.825 |  | 510.50 |  |  |
| 74 | 3046 |  | 3046 | 73.47 | 1.019 |  | 388.67 |  |  |
| 75 | 3286 |  | 3286 | 80.74 | 1.433 |  | 216.69 |  |  |
| 76 | 3172 |  | 3172 | 77.29 | 1.225 |  | 290.75 |  |  |
| 77 | 2380 |  | 2380 | 53.30 | 0.132 |  | 1355.81 |  |  |
| 78 | 3292 |  | 3292 | 80.92 | 1.445 |  | 213.13 |  |  |
| 79 | 2656 |  | 2656 | 61.66 | 0.475 |  | 836.17 |  |  |
| 80 | 2741 |  | 2741 | 64.23 | 0.586 |  | 715.66 |  |  |
| 81 | 3394 |  | 3394 | 84.01 | 1.659 |  | 157.61 |  |  |
| 82 | 2494 |  | 2494 | 56.75 | 0.272 |  | 1113.68 |  |  |
| 83 | 2540 |  | 2540 | 58.15 | 0.329 |  | 1027.71 |  |  |
| 84 | 2552 |  | 2552 | 58.51 | 0.344 |  | 1006.28 |  |  |
| 85 | 3273 |  | 3273 | 80.35 | 1.408 |  | 224.50 |  |  |
| 86 | 2855 |  | 2855 | 67.69 | 0.739 |  | 576.13 |  |  |
| 87 | 2977 |  | 2977 | 71.38 | 0.914 |  | 450.46 |  |  |
| 88 | 3264 |  | 3264 | 80.07 | 1.391 |  | 230.00 |  |  |
| 89 | 3313 |  | 3313 | 81.56 | 1.487 |  | 200.96 |  |  |
| 90 | 3023 |  | 3023 | 72.77 | 0.983 |  | 408.58 |  |  |
