# Supplementary figures and images for: AANAT transgenic sheep generated via OPS vitrified-microinjected pronuclear embryos and reproduction efficiency of the transgenic offspring
Source: PeerJ. 2018 Aug 8;6:e5420. doi: 10.7717/peerj.5420 (PMC6087419; doi:10.7717/peerj.5420)

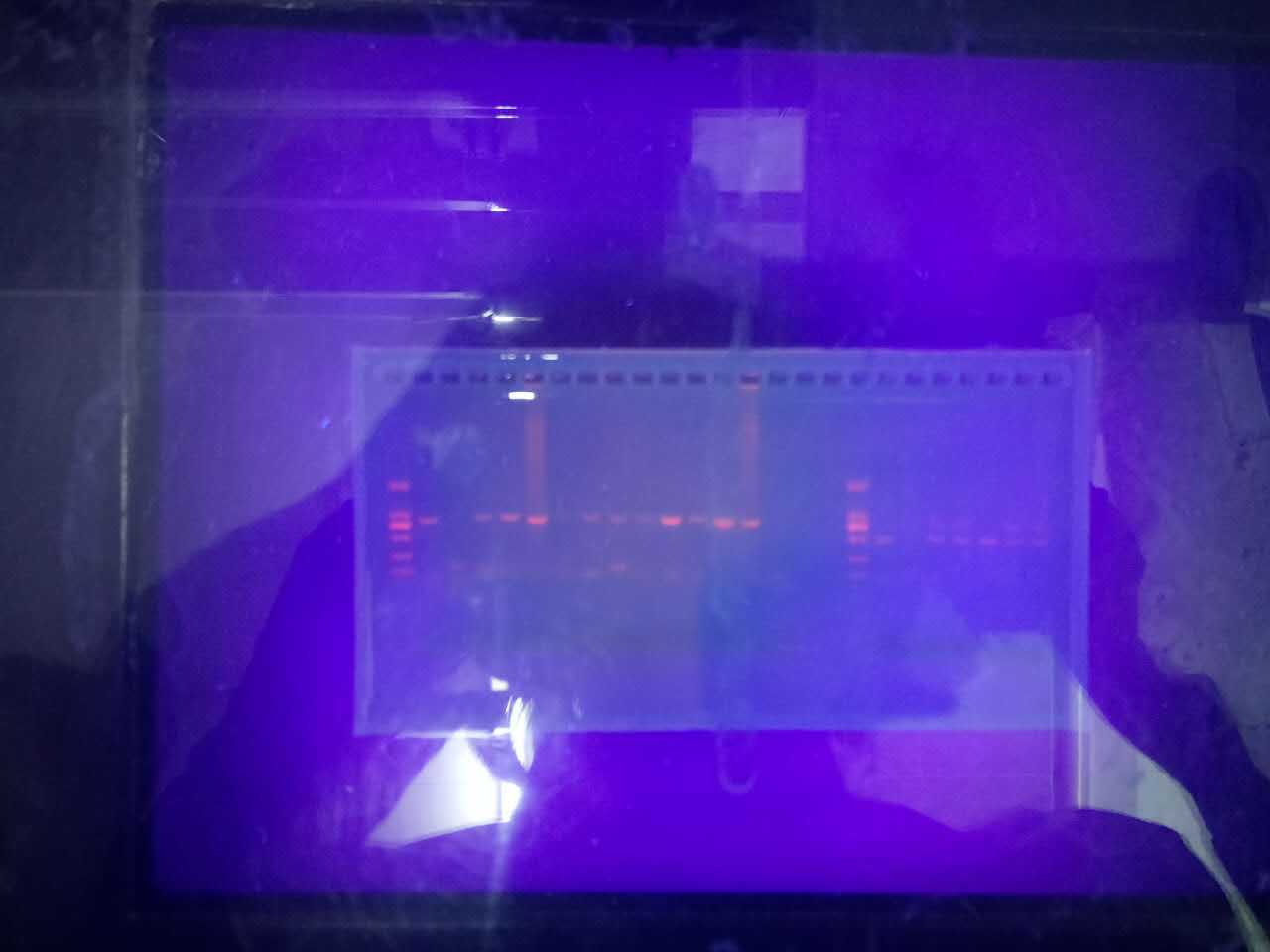

Supplement: Supplemental Information 1 [file peerj-06-5420-s001.zip › Raw data/PCR figure.jpg]

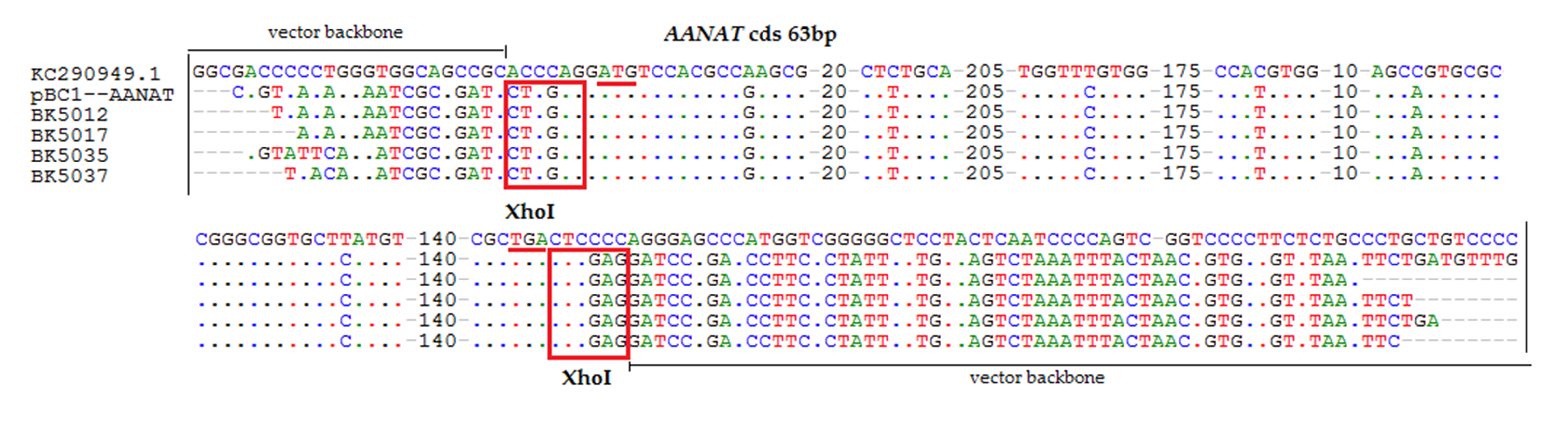

Supplement: Supplemental Information 1 [file peerj-06-5420-s001.zip › Raw data/Sequence diagram.jpg]

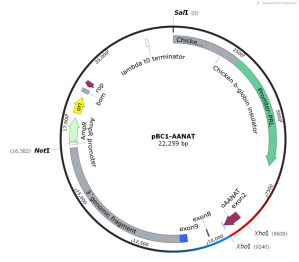

Supplement: Supplemental Information 1 [file peerj-06-5420-s001.zip › Raw data/vector construction.tif]

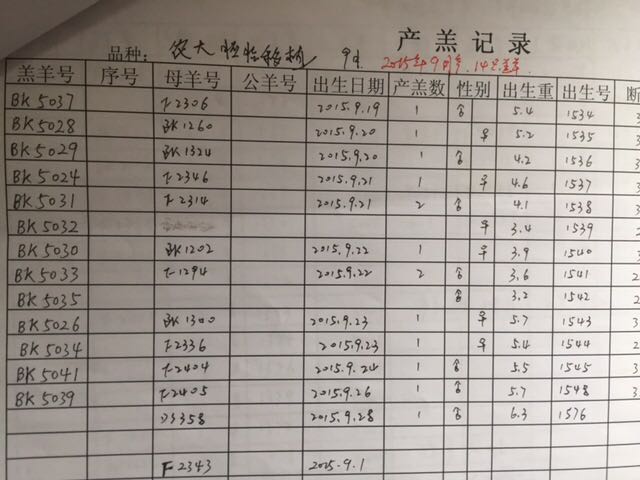

Supplement: Supplemental Information 1 [file peerj-06-5420-s001.zip › Raw data/The serial number of the sheep.jpg]
